# Focal adhesion ribonucleoprotein complex proteins are major humoral cancer antigens and targets in autoimmune diseases

**DOI:** 10.1038/s42003-020-01305-5

**Published:** 2020-10-16

**Authors:** Shinichiro Atsumi, Hiroto Katoh, Daisuke Komura, Itaru Hashimoto, Genta Furuya, Hirotomo Koda, Hiroki Konishi, Ryohei Suzuki, Asami Yamamoto, Satsuki Yuba, Hiroyuki Abe, Yasushi Rino, Takashi Oshima, Tetsuo Ushiku, Masashi Fukayama, Yasuyuki Seto, Shumpei Ishikawa

**Affiliations:** 1 Department of Preventive Medicine, Graduate School of Medicine, the University of Tokyo, Tokyo, Japan; 2 Department of Gastrointestinal Surgery, Graduate School of Medicine, the University of Tokyo, Tokyo, Japan; 3 Department of Surgery, Yokohama City University, Kanagawa, Japan; 4 Department of Pathology, Graduate School of Medicine, the University of Tokyo, Tokyo, Japan; 5 Department of Molecular Pathology, Graduate School of Medical and Dental Sciences, Tokyo Medical and Dental University, Tokyo, Japan; 6 Department of Gastrointestinal Surgery, Kanagawa Cancer Center, Kanagawa, Japan

**Keywords:** Tumour immunology, Cancer microenvironment

## Abstract

Despite the accumulating evidences of the significance of humoral cancer immunity, its molecular mechanisms have largely remained elusive. Here we show that B-cell repertoire sequencing of 102 clinical gastric cancers and molecular biological analyses unexpectedly reveal that the major humoral cancer antigens are not case-specific neo-antigens but are rather commonly identified as ribonucleoproteins (RNPs) in the focal adhesion complex. These common antigens are shared as autoantigens with multiple autoimmune diseases, suggesting a direct molecular link between cancer- and auto-immunity on the focal adhesion RNP complex. This complex is partially exposed to the outside of cancer cell surfaces, which directly evokes humoral immunity and enables functional bindings of antibodies to cancer cell surfaces in physiological conditions. These findings shed light on humoral cancer immunity in that it commonly targets cellular components fundamental for cytoskeletal integrity and cell movement, pointing to a novel modality of immunotherapy using humoral immunological reactions to cancers.

## Abstract (web summary)

Atsumi et al. identify antigens to B-cell receptors isolated from gastric tumour samples. They find that focal adhesion-related protein complexes, several of which are also targets for autoimmune disease, are major humoral cancer antigens. These findings provide insights into humoral immunity in tumor environments.

## Introduction

Recently, anti-tumor immunotherapies aiming to activate the cellular immunity of T cells using anti-PD-1/PD-L1 or CTLA-4 antibodies have attracted attention thanks to their highly effective and long-lasting clinical outcomes;^1^ thus, in-depth investigations of the molecular mechanisms of T cell immunity have been carried out to expand our understanding of anti-tumor T cell immunity. In contrast, although several lines of increasing evidence of in vitro, in vivo, and clinical settings have shown the significant role of humoral immunity in tumors played by B cells^2,3^, its precise molecular mechanism has largely been obscure. For example, the kinds of tumor antigens the tumor-infiltrating B cells recognize or the common biological properties, if any, of the humoral tumor antigens have not yet been clarified. To make B cell immunity applicable as a therapeutic modality, it is essential to deepen our understanding of tumoral B cell immunity.

Gastric cancer (GC) is one of the most frequent malignancies and has one of the worst prognoses worldwide, especially in east-Asian countries^4^. Clinical trials of checkpoint inhibitors against GCs revealed that GCs did not benefit from current immunotherapies^5^, with some exceptions in which highly efficacious responses were observed in microsatellite instability-type GCs as expected^6,7^ and Epstein-Barr virus (EBV)-associated GCs. Therefore, it is necessary to establish novel strategies of immunotherapies against GCs from standpoints other than T cell immunity; in this context, one possible strategy might be to utilize humoral immunity.

Every single B cell expresses specific B cell receptors (BCRs) (immunoglobulins when secreted) consisting of more than 10^18^ repertoires in individuals, via genetic recombination of complexed immunoglobulin gene loci^8^. In a previous report, we described a global immunogenetic picture of tumor-infiltrating B cell repertoires in 30 GCs, discovering dominantly expanded B cell clones in tumor environments in some cases^9^. Biochemical analyses of reconstructed human immunoglobulin G (IgGs) of the dominant B cell clones enabled us to identify their corresponding antigens. Some of the IgGs unexpectedly and commonly recognized sulfated-glycosaminoglycans, and others reacted to protein antigens, such as EZRIN (EZR), heat shock protein 90 (HSP90), and Lamin A (LMNA)^9^. Thus, our immunogenomic approach was a useful strategy to identify humoral tumor antigens. However, the precise biological and clinical significance shared among those identified protein antigens has been unclear due to the paucity in numbers of identified protein antigens. A straightforward strategy to obtain common features of humoral protein antigens in tumors is to perform immunogenomic analysis for a larger cohort of GCs and to expand the catalog of humoral tumor antigens, which further deepens the understanding of humoral tumor immunity and helps come up with novel anti-tumor immunotherapies. In this study, our immunogenetic investigation of 102 cases of GC reveals previously unknown and intriguing common features of humoral antigens in tumor environments.

## Results

### Identification of tumor-specific dominant B cell clonotypes in 102 GCs and Biochemical screening for their humoral antigens

**Fig. 1:**
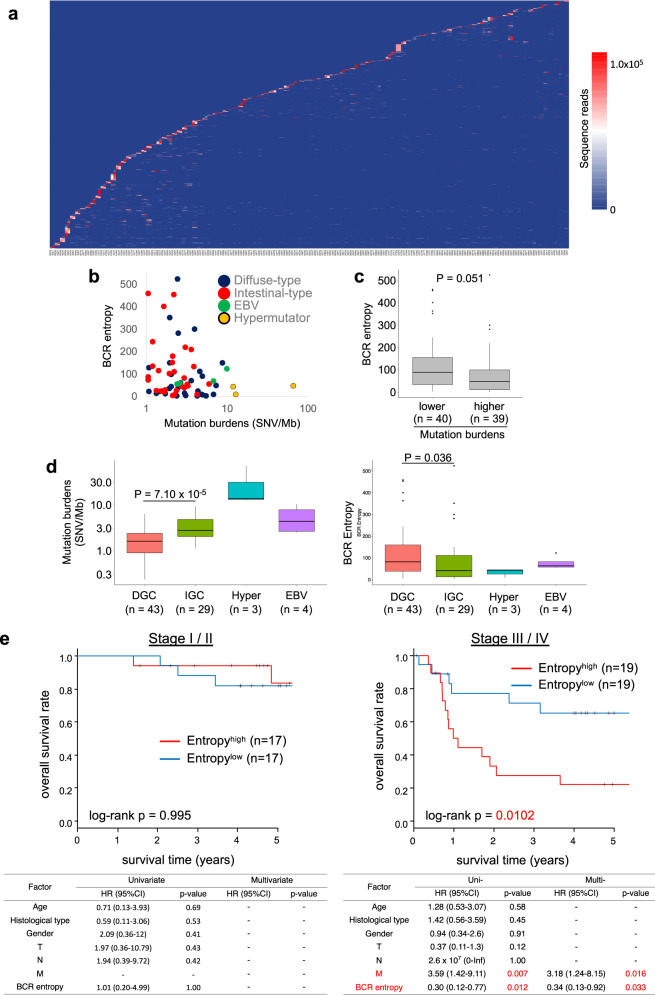
B cell receptor (BCR)/immunoglobulin repertoire profiles and clinicopathological factors. **a** Shared CDR3 amino acid sequences across 102 GC individuals. A total of 102 GC cases (N/T) are plotted along the *x* axis; then, unique CDR3s with more than 5000 sequence reads among the 102 cases (N + T) are sorted in ascending lexicographical order based on the number of sequence reads in each sample. The color scale indicates the number of sequence reads. Cases #1 to #30 were analyzed in our previous study^9^. **b** BCR entropy (*y*-axis) is plotted according to the mutation burdens of the GCs (*x*-axis). Colors indicate GC subtypes, as indicated. Mutation burdens and hypermutator GCs were defined in our previous report^10^. EBV, Epstein-Barr virus-associated GC. **c** The *y*-axis indicates the BCR entropy in GC groups with lower and higher mutation burdens as separated by the median of mutation burdens (2.22 SNV/Mb). Center line, median; box limits, upper and lower quartiles; whiskers, 1.5× interquartile range; dots, outliers. *P*-value was calculated using the Mann–Whitney *U* test. **d** Mutation burdens (left) and BCR entropies (right) are plotted according to the GC subgroups, as indicated. DGC: diffuse-type GC; IGC: intestinal-type GC; Hyper: hypermutator. Ns indicate the number of cases. Center line, median; box limits, upper and lower quartiles; whiskers, 1.5× interquartile range; dots, outliers. *P*-values were calculated using the Mann–Whitney *U* test. **e** The overall clinical outcomes of the GC patients were analyzed in terms of BCR entropies in the tumor environments. The Kaplan–Meier method indicated that GC cases with BCR entropies higher than the median (88.5 and 54.5 for stage I/II and stage III/IV cases, respectively) exhibited significantly worse prognosis among stage III/IV advanced cases. Multivariate analysis with backward stepwise selection revealed that BCR entropy, together with M classification, is an independent prognostic factor of the overall survival of patients with advanced GC.

Clinicopathological data of the patients analyzed in this study are summarized in Supplementary Data 1 and 2. It was revealed that overall BCR repertoire profiles differ substantially between patients (Fig. 1a), confirming the extra-high diversity of the BCR repertoires in our body^9^. It was found that higher whole-genomic mutation burdens, as defined in our previous study^10^, had an apparent tendency to be correlated with lower BCR entropy (higher clonality of B cells) among various subtypes of GCs (Fig. 1b–d). Furthermore, it was noted that BCR entropy in tumor environments is an independent prognostic factor for advanced stage GC (Fig. 1e).

**Fig. 2:**
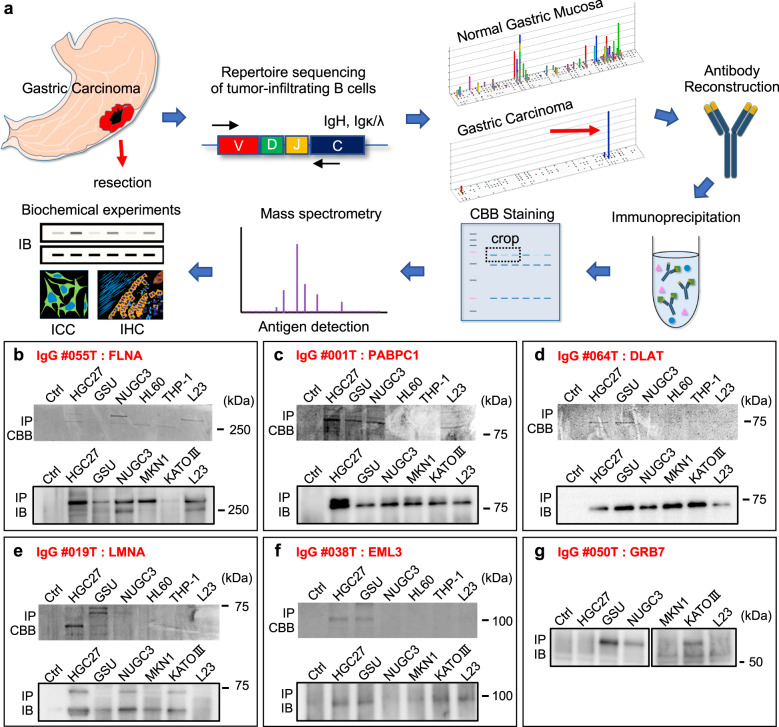
Identification of protein antigens for tumor-specific dominant B cell clones. **a** A schematic summary of our screening strategy. Following the discovery of tumor-specific and dominant chronotypes of tumor-infiltrating B cells by repertoire sequencing, their protein antigens were biochemically explored. **b**–**g** All the protein antigens identified in this study are shown. The upper panels show Coomassie Brilliant Blue (CBB)-stained SDS-PAGE results of immunoprecipitation (IP) using reconstructed human immunoglobulins. The lower panels show confirmative immunoblots of immunoprecipitated lysates of independent IP experiments using commercial antibodies. HGC27, GSU, NUGC3, MKN1, and KATOIII are human GC cell lines; HL60 and THP-1 are human promyelocytic leukemia cell and leukemic monocyte, respectively; and L23immo is an immortalized human fibroblast cell line. “Ctrl” represents the immunoglobulin-only lane. IP experiments were performed at least twice.

Our experimental procedure is summarized in Fig. 2a. We initially performed BCR/immunoglobulin repertoire sequencing of tumor-infiltrating B cells in 102 GC cases. BCRs/immunoglobulins comprise hetero-tetramers of two molecules; namely, heavy chains (IgA, D, E, M, and G) and light chains (Ig κ and λ). We sequenced nearly the full lengths of both of these two repertoires, as described previously^9^. In most of the GC cases, we could obtain substantial numbers of tumor-specific immunoglobulin heavy and light chain sequences, which prompted us to reconstruct those immunoglobulins to investigate their humoral tumor antigens. Since we sequenced the heavy and light chain repertoires in two independent reactions^9^, it was necessary to define the proper combinations of tumor-specific heavy and light chains from those separate repertoire data. To this end, we further searched GC cases in which both the heavy and light chains simultaneously and apparently dominated in the tumor samples. In most cases, only the top-ranked dominant clonotypes of the heavy and light chains were combined and reconstructed as immunoglobulins (Supplementary Data 3 and Supplementary Fig. 1). The relevance of this strategy to define the combinations of heavy and light chains was validated in our previous report^9^. In the present study, we discovered 26 tumor-specific and dominant B cell clones from the repertoire data (Supplementary Data 3 and Supplementary Fig. 1).

Protein antigen screening was then carried out to identify the corresponding antigens of the 26 reconstructed human antibodies (Fig. 2a and Supplementary Fig. 2). First, immunoprecipitation (IP) experiments were performed using human cell line lysates for each antibody to identify the candidate protein antigens. To widen the diversity of the screening targets, we utilized various lineages of cell lines for IP to include GC, hematopoietic, and fibroblast cells (Fig. 2b–f). All IP experiments were conducted using a set of various antibodies simultaneously and only specific protein bands in each antibody were analyzed by mass spectrometry (MS). This IP/MS screening identified five protein antigens (filamin A [FLNA], poly-A binding protein cytoplasmic 1 [PABPC1], dihydrolipoamide S-acetyltransferase [DLAT], LMNA, and EMAP-like 3 [EML3]) (Supplementary Data 4), which were confirmed by immunoblots (Fig. 2b–f); in total, seven antigens for eight antibodies were discovered, including EZR and HSP90 reported in our previous study^9^. We further tried to identify the protein antigens of the other orphan antibodies by protein array (HuProt®) and identified an additional protein antigen, growth factor receptor-bound protein 7 (GRB7) (Fig. 2g and Supplementary Figs. 2 and 3). Finally, we obtained eight protein antigens for nine reconstructed antibodies. The other 17 antibodies may react to non-protein antigens, such as sulfated-glycosaminoglycan, another major humoral antigen in cancer environments^9^. Whole-exome sequencing (WES) revealed that none of the identified protein antigens were somatically mutated in the GC cases in which the immunoglobulins were originally discovered (data is available from our previous report^10^); therefore, the identified protein antigens were not case-specific “neo-antigens” but rather were usual common cellular proteins.

### Identified humoral protein antigens in tumor microenvironments are also frequent targets of various autoimmune diseases

We next investigated the common features of the identified protein antigens of the tumor-specific dominant B cell clones. Knowledge-based exploration of the biological and clinical features of the protein antigens revealed that almost all the humoral protein antigens we discovered in tumor environments have also been reported as immunological targets of humoral autoimmunity in multiple autoimmune diseases such as systemic lupus erythematosus (SLE), rheumatoid arthritis (RA), myasthenia gravis (MG), multiple sclerosis (MS), primary biliary cirrhosis (PBC), and Sjogren’s syndrome (SS)^11–22^ (Table 1). This shared feature of humoral immunity in tumors and autoimmunity in view of their target protein antigens suggested that these two clinically important aspects of immunological dysregulations are attributable to common molecular mechanisms.

**Table 1:** Identified protein antigens and knowledge-based investigation of their interactions with autoimmune diseases.

Antigens	Full name	Identified as an autoantigen in autoimmune diseases	Reference
FLNA	Filamin A	Rheumatoid arthritis	Pianta et al.^11^
		Myasthenia gravis	Yamamoto et al.^12^
DLAT	Dihydrolipoamide S-acetyltransferase	Primary biliary cirrhosis	Van de Water et al.^13^
PABPC1	Poly-A binding protein cytoplasmic 1	Myasthenia gravis	Becker et al.^14^
GRB7	Growth factor receptor bound protein 7	Rheumatoid arthritis	Choong et al.^15^
LMNA	Lamin A	Primary biliary cirrhosis	Hu et al.^16^
		Rheumatoid arthritis	Lassoued et al.^17^
		Sjogren’s syndrome	Zhang et al.^18^
HSP90^a^	Heat shock protein 90	Systemic lupus erythematosus	Minota et al.^19^
		Rheumatoid arthritis	Mantej et al.^20^
		Multiple sclerosis	Cid et al.^21^
EZR^a^	Ezrin	Rheumatoid arthritis	Wagatsuma et al.^22^
EML3	EMAP like 3	Not identified yet	

^a^Ezrin and HSP90 were identified in our previous study (Katoh et al.^9^).

### Focal adhesion-related protein complexes are major and common targets of humoral immunity in tumor microenvironments

**Fig. 3:**
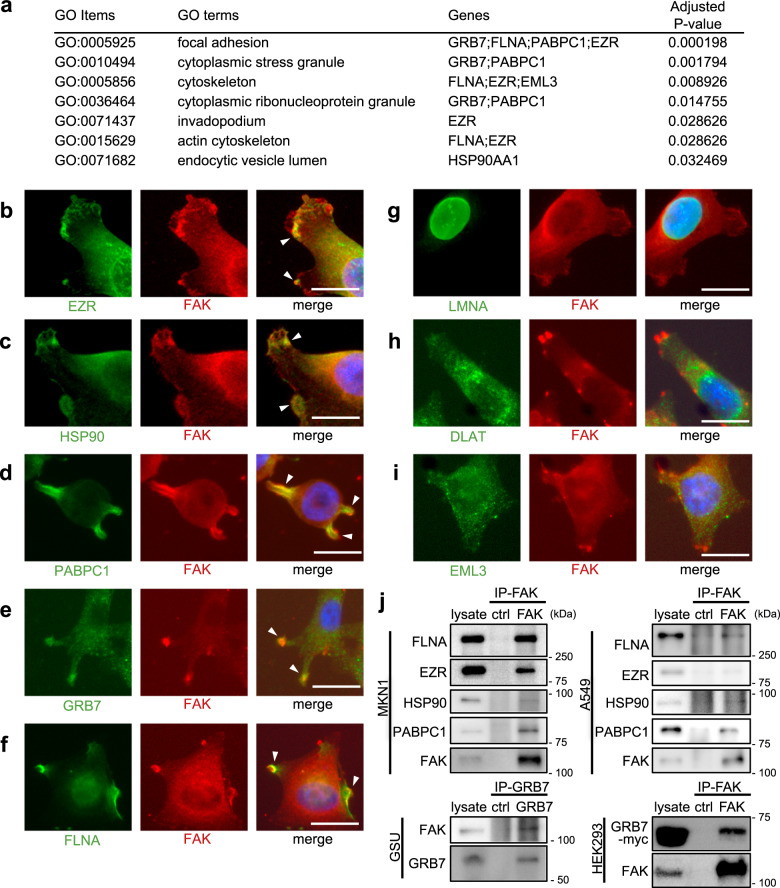
Focal adhesion-related protein complexes are major and common humoral antigens in tumor microenvironments. **a** GO enrichment analysis of the eight identified protein antigens. **b**–**i** Fluorescent immunocytochemistry of the identified protein antigens (green) along with focal adhesion kinase (FAK) (red) onto a human gastric cancer cell line MKN1. The blue color represents Hoechst nuclear staining. The white bars indicate 20 μm. The white arrowheads indicate representative hot spots of colocalization between protein antigens and focal adhesions (only in **b**–**f**). Similar colocalizations were reproducibly observed in all analyzed cells with formations of visible focal adhesions (only in **b**–**f**). The same immunocytochemical staining was also performed onto another human gastric cancer cell line GSU, obtaining similar results (Supplementary Fig. 4). **j** Co-immunoprecipitation experiments of the FAK complex. The upper and lower panels at the left side show co-IP using anti-FAK antibody for MKN1 and anti-GRB7 antibody for GSU, respectively, followed by immunoblots using the indicated antibodies. The right-side panels show co-IP using anti-FAK antibody for A549, a lung adenocarcinoma cell line, and HEK293 transfected with GRB7-myc/His construct, respectively, followed by immunoblots using the indicated antibodies. Co-IP experiments were conducted at least twice.

Gene ontology (GO) enrichment analysis of our eight identified protein antigens revealed that the most enriched “cellular component” category was “focal adhesion” (Fig. 3a). In this GO enrichment analysis, four proteins (GRB7, FLNA, PABPC1, and EZR) were assigned localizations to focal adhesion. In order to test this hypothetical enrichment of protein antigens in the focal adhesion machinery, immunocytochemical dual staining was carried out for all the protein antigens we discovered, together with focal adhesion kinase (FAK), a focal adhesion marker (Fig. 3b–i and Supplementary Fig. 4). The results validated that the focal adhesion-related proteins (GRB7, FLNA, PABPC1, and EZR) significantly co-localized with FAK (Fig. 3b, d, e, and f, and Supplementary Fig. 4); moreover, HSP90 also exhibited dense deposits at the focal adhesion sites (Fig. 3c and Supplementary Fig. 4). Not merely GC cells but also other cancer cell lines such as from lung and pancreatic adenocarcinomas also exhibited clear co-localizations between FAK and the protein antigens (Supplementary Fig. 5). Co-localizations of these protein antigens with FAK were robustly observed in all analyzed cells with formations of visible focal adhesions. These observations were not non-specific phenomena in our experimental setting, since co-localizations of FAK and other protein antigens, LMNA, DLAT, and EML3, were not clearly observed at the focal adhesions in the cells analyzed (Fig. 3g–i and Supplementary Fig. 4). Not only co-localizations but also physical interactions of FAK and the protein antigens were proved in co-IP experiments. Co-IP of MKN1 and A549 cell lysate using an anti-FAK antibody showed that FLNA, HSP90, PABPC1, and EZR physically co-precipitated with FAK (Fig. 3j upper panels). GRB7 was not clearly observed in the FAK-IP in our experimental settings, probably due to the low IP efficiency of the FAK antibody we used or a lower expression level of the GRB7 protein in cell lines; however, a counterpart GRB7-IP using GSU cells and an FAK-IP using GRB7-overexpressed HEK293 cells robustly showed physical interactions between GRB7 and FAK (Fig. 3j lower panels). Together, these findings demonstrated that focal adhesion-related protein complexes are major and common humoral antigens of B cell immunity in tumors.

**Fig. 4:**
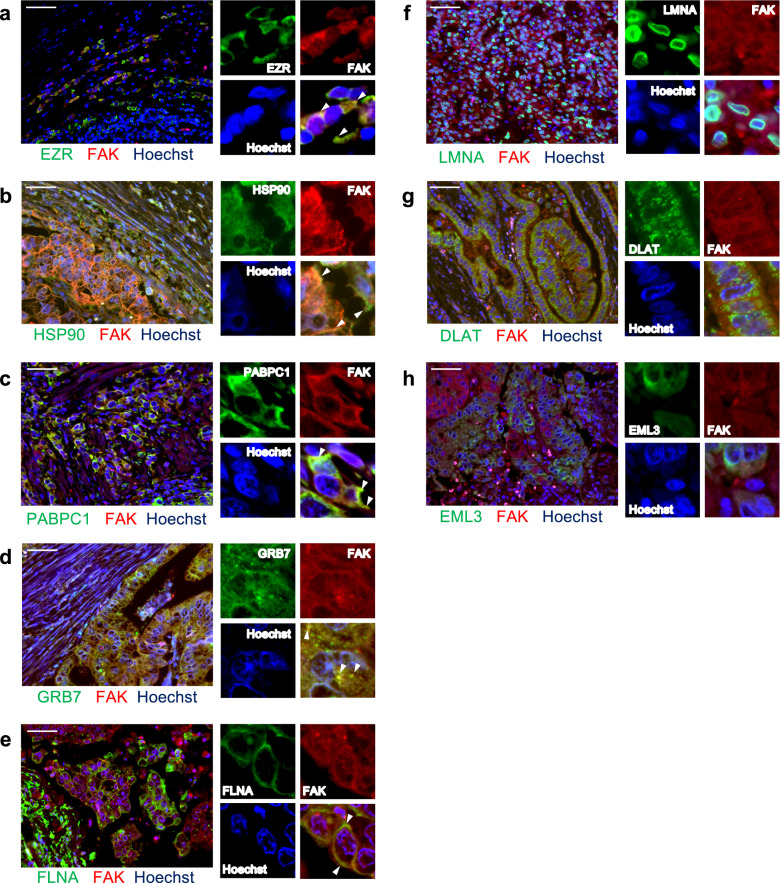
Co-localizations of the identified protein antigens with focal adhesions in clinical specimens. **a**–**h** Fluorescent immunohistochemistry of the identified protein antigens (green) along with FAK (red) was performed onto the clinical GC specimens from which these immunoglobulins were originally discovered. The blue color represents Hoechst for nuclear staining. The white bars indicate 50 μm. The white arrowheads indicate representative colocalization of protein antigens and focal adhesions in cancer cells (only in **a**–**e**). Clear colocalizations were reproducibly observed in multiple of GC cells in at least five randomly selected microscopic fields in these clinical GC samples (only in **a**–**e**).

We also performed immunofluorescent dual staining of the clinical GC specimens from which the reconstructed immunoglobulins were originally discovered (Fig. 4a–h). The focal adhesion structures were, in most cases, hardly identifiable in clinical specimens; however, close observations of immunofluorescent staining validated the co-localizations of FAK and five protein antigens (GRB7, FLNA, HSP90, PABPC1, and EZR) in these clinical tumor tissues. Such co-localizations between FAK and the identified protein antigens in clinical specimens were also observed in other malignancies such as lung and colorectal adenocarcinomas (Supplementary Fig. 6); implying that these co-localizations are general phenomena across various cancers. Immunohistochemical evaluation of immune cells in the tumors from which the FAK-related protein antigens were discovered showed that infiltrating B cells were, in general, in their activated states in these GC cases (Supplementary Fig. 7).

### The focal adhesion-related humoral antigens are ribonucleoproteins

**Fig. 5:**
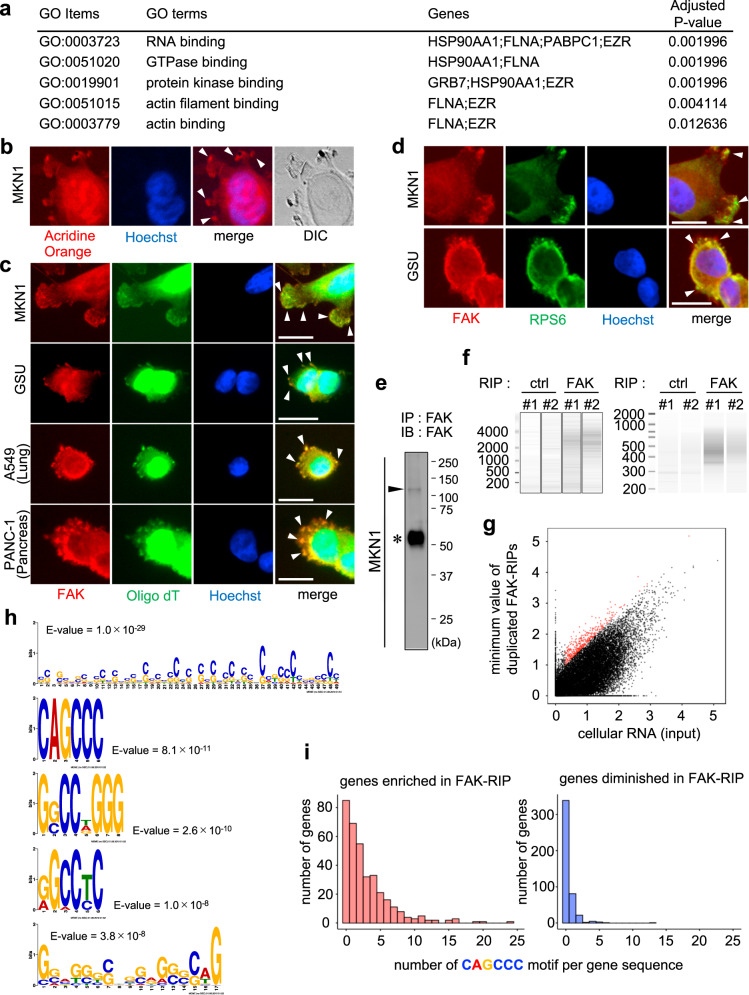
The identified humoral antigens in the focal adhesion complexes are ribonucleoproteins (RNPs) colocalized with ribosomal translation machinery. **a** GO enrichment analysis of the five protein antigens correlated with focal adhesion complexes. **b** Fluorescent staining of single-strand nucleotides using acridine orange in MKN1 cells. The blue color represents Hoechst nuclear staining. DIC, differential interference contrast image. **c** Fluorescent dual staining of anti-FAK antibody (red) and Cy5-conjugated oligo-dT probe (green) in human gastric cancer cells MKN1 and GSU, a lung adenocarcinoma cell A549, and a pancreatic cancer cell PANC-1. The blue color represents Hoechst staining. The white bars indicate 20 μm. **d** Fluorescent immunocytochemistry of FAK (red) and a ribosome marker (ribosomal protein S6 [RPS6]) (green). The blue represents nuclear staining. The white bars indicate 20 μm. **b**–**d** White arrowheads indicate representative colocalization signals, and the similar colocalizations were reproducibly observed in all analyzed cells with formations of visible focal adhesions. **e** The validity of the anti-FAK antibody in our RIP procedure was confirmed by immunoblots. The arrowhead and asterisk represent the FAK protein and heavy chain of the anti-FAK rabbit antibody, respectively. **f** The left and right panels show electropherograms of the precipitated RNA and constructed cDNA sequencing library, respectively, of FAK-RIP experiments in biological duplicates (#1 and #2). **g** Plots of the results of the next-generation sequencing of FAK-RIP. The *x* and *y*-axes represent the numbers of sequencing reads of input cellular RNA and the minimum of those in the two FAK-RIP experiments, respectively. Each dot represents each transcript/gene, while red dots indicate the enriched transcripts/genes used in the motif enrichment analysis. **h** The top five enriched motifs in the transcript sequences among FAK-RIP-enriched genes (354 genes) compared to FAK-RIP-diminished genes (454 genes). **i** Histograms of the numbers of genes plotted according to the numbers of “CAGCCC” motifs within the gene transcript sequences. The left and right panels represent FAK-RIP-enriched and -diminished gene sets, respectively.

The focal adhesion-related protein antigens identified above were further subjected to GO enrichment analysis focusing on “molecular function”. In this analysis, the most highly enriched category was “RNA-binding” function (Fig. 5a). Four of the five focal adhesion-related protein antigens—HSP90, FLNA, PABPC1, and EZR—were categorized as RNA-binding proteins; moreover, GRB7 is also known as an RNA-binding protein^23^. Based on these findings, we hypothesized that the focal adhesion-related humoral tumor antigens we discovered are ribonucleoproteins (RNPs). To validate this hypothesis, we performed cellular fluorescent staining using a single-strand nucleotide-specific probe, acridine orange, in the MKN1 GC cell line, which forms clear focal adhesions, and confirmed dense localizations of RNAs at the focal adhesion sites (Fig. 5b). Fluorescent dual staining of the Cy5-conjugated oligo-dT probe and anti-FAK antibody further proved the co-localization of poly(A)-containing RNAs and focal adhesions (Fig. 5c), which were also confirmed in lung and pancreatic adenocarcinoma cell lines (Fig. 5c). Moreover, our dual-color immunocytochemical analysis of FAK and RSP6, a ribosome marker, clearly exhibited co-localization of ribosomal translational machinery with focal adhesions (Fig. 5d). Thus, focal adhesions work as anchors of RNPs to form on-site translational processing bodies^24^.

These findings support the conclusion that humoral immunity in tumors commonly targets focal adhesion-related RNP complexes. This finding is of significance since RNPs are well-known triggers of B cell activation and/or proliferation via the Toll-like receptor (TLR) signaling axis^25–27^, which may be linked to a mechanism of the dominant expansion of B cells against focal adhesion-related RNPs, as discussed later. This intriguing finding prompted us to perform RNA-precipitation (RIP) to investigate the molecular biological significance of the focal adhesion-bound RNAs. Using the focal adhesion-rich GC cell line MKN1, we performed RIP with an anti-FAK antibody (FAK-RIP) (Fig. 5e–i, Supplementary Fig. 8 and Supplementary Data 5–7). The FAK-RIP confirmed that RNA molecules were abundant at focal adhesions (Fig. 5f), and we successfully identified 354 genes/transcripts that were reproducibly enriched in the biological replicates of FAK-RIP precipitates (Fig. 5g, Supplementary Fig. 8, and Supplementary Data 5 and 6). Motif enrichment analysis of the FAK-RIP-precipitated RNAs identified a variety of motifs including non-specific G/C-rich sequences as well as specific motifs, such as CAGCCC (Fig. 5h). CAGCCC was significantly enriched in both entire gene sequences and 3′ UTRs (Fig. 5i and Supplementary Fig. 8d) and was more frequent compared to its permutated and their opposite-strand motifs (Supplementary Fig. 8e,f). The results of these bioinformatic investigations suggested that focal adhesions contain specific sets of genes/transcripts and that focal adhesion-related RNPs may serve as transporters of RNAs harboring specific motifs such as CAGCCC and its similar ones.

### Focal adhesion complexes are topologically exposed to cellular surfaces and can be direct targets of B cells and antibodies in physiological conditions

**Fig. 6:**
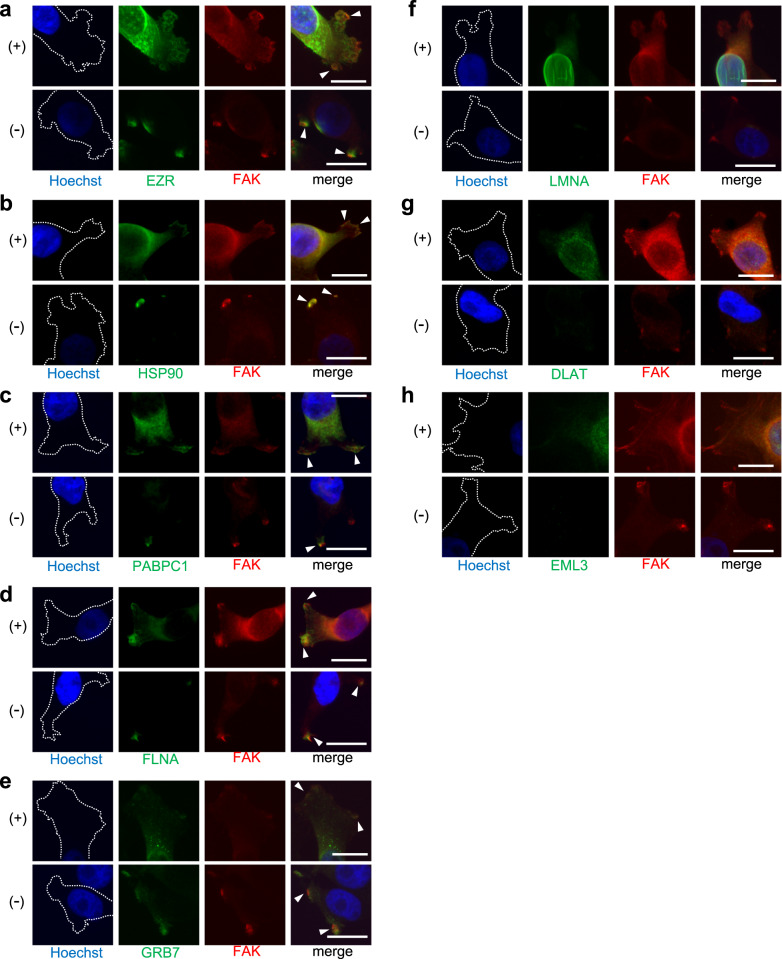
Focal adhesion complexes are occasionally topologically exposed on the outside of cellular membranes and can be targeted directly by B cell immunity. **a**–**h** Fluorescent dual staining of the identified protein antigens (green) and FAK (red) in MKN1 cells. The cells were either permeabilized (+) or non-permeabilized (−) before immunostaining. The blue color represents Hoechst nuclear staining. The white bars indicate 20 μm. The white arrowheads represent hotspots of colocalizations of protein antigens with FAK. In the permeabilization (+) experiments, colocalizations were reproducibly observed in all analyzed cells with the formations of visible focal adhesions (only in **a**–**e**). In the permeabilization (−) experiments, colocalizations were robustly observed in all the investigated cells in which clear FAK signals (red) were detected at visible focal adhesion sites (only in **a**–**e**).

Next, we assessed the mechanisms by which B cells react to such focal adhesion complex proteins. B cells, unlike T cells, recognize only exposed antigens; thus, intracellular proteins cannot be the direct targets of B cell immunity if they are not released to the outside of cells such as by cell death. However, in certain situations, cytosolic sub-membranous proteins can be exposed to cellular surfaces;^28,29^ therefore, we hypothesized that focal adhesion complexes can also be exposed to cell surfaces under physiological conditions. To test this hypothesis, we conducted dual-color immunocytochemistry using antibodies to FAK and the identified protein antigens in the MKN1 cell line, either with or without permeabilization (Fig. 6a–h). The results showed that FAK complex proteins, including FAK protein itself, were topologically exposed to the outside of cellular membrane (Fig. 6a–e). The unrelated protein antigens (LMNA, DLAT, and EML3) did not show such exposures to the cell surfaces (Fig. 6f–h). Immunohistochemical staining was also performed for clinical GC specimens, and it was suggested that the FAK complex, on occasions, might be exposed to the outer spaces of the cellular membrane (Supplementary Fig. 9), although such an analysis of protein topology is difficult to conduct for clinical specimens. Therefore, the focal adhesion machinery is at least occasionally exposed to the outer space of cell surface and can be directly recognized by B cells that trigger active humoral immunity, as well as by secreted antibodies.

## Discussion

**Fig. 7:**
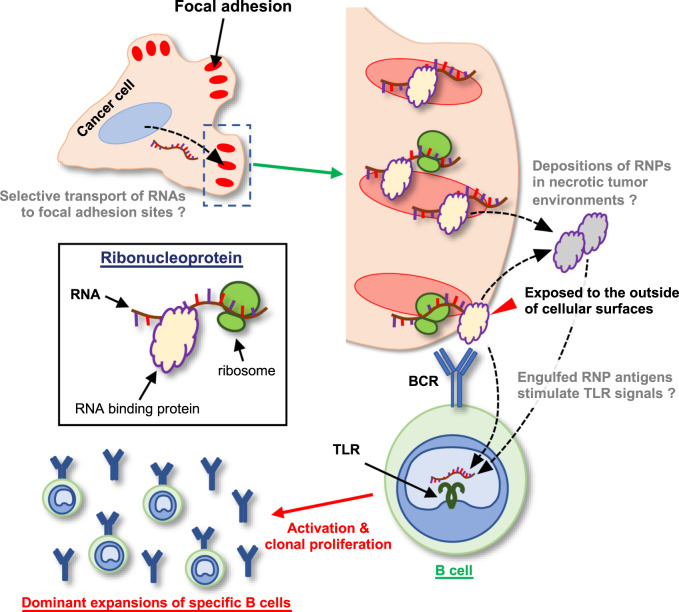
A schematic summary of our findings that focal adhesion-related RNPs are frequent and common humoral antigens in tumor microenvironments. Focal adhesion machineries complexed with ribonucleoproteins and RNA molecules are frequent humoral antigens in tumor environments. This study suggested sequence-specific trafficking of RNA molecules to focal adhesion sites in cancer cells. Such focal adhesion complexes are occasionally exposed to the outside of cancer cells (red arrowhead) and/or may be broken down and deposited in necrotic cancer stromal environments (gray RNPs); thus, they are considered to trigger and expand B cell immunity in cancer microenvironments. TLR, Toll-like receptors; RNP, ribonucleoproteins.

The results of this study revealed previously unknown aspects of humoral tumor immunity following large-scale immunogenomic analysis of tumor-infiltrating B cells in a large cohort of clinical GC cases. We found that focal adhesion-related RNP complexes are common major humoral antigens in tumor microenvironments (Fig. 7), most of which are also common targets of autoimmune diseases such as SLE, RA, MG, MS, PBC, and SS (Table 1). Our data not only clarified a general phenomenon of humoral immunity in human cancers but also proposed a hypothetical new axis of autoimmunity, both of which are directly linked by the RNP complex in the focal adhesion machinery. It is also of note that the identified protein antigens are not case-specific “neo-antigens” but rather are common cellular proteins; therefore, these humoral protein antigens are commonly shared among wide varieties of malignancies regardless of their somatic mutation profiles.

This intriguing enrichment of the humoral protein antigens in RNPs of the focal adhesion complex may indicate the presence of a signaling pathway that underlies the dominant expansion of B cells in tumor environments. It is well known that humoral immunity can be activated and/or enhanced by the TLR signaling axis in B cells^25,26^. TLR3 and TLR7/8, for instance, recognize ribonucleotide molecules, such as dsRNA and/or ssRNA^27^ to induce activating signals in antigen-bound B cells to proliferate in situations in which their protein antigens exist as complexed forms with ribonucleotides; in other words, as RNPs^25,26^. This fact is compatible with our finding of the enrichment of humoral tumor antigens in the focal adhesion-related RNPs in that, once the antigen RNPs were bound by and engulfed into B cells, those B cells are cell-autonomously activated by endogenous downstream signals of TLRs, resulting in the dominant expansion of the B cells in the tumor microenvironments (Fig. 7). It is necessary to confirm this hypothesis through further experimentation.

The FAK-RIP experiment targeting the endogenous focal adhesion complex further confirmed the enrichment of RNAs in the focal adhesion sites (Fig. 5 and Supplementary Fig. 8). Previous reports also showed the enrichment of mRNAs/ribosomes at focal adhesions and lamellipodium structures;^30–32^ however, our data showing biased enrichment of specific RNAs further indicated that the FAK complex plays a role as a cargo of RNAs that transports specific RNAs such as those with a CAGCCC motif, for instance, to the focal adhesion machinery (Fig. 5 and Supplementary Fig. 8). Although not statistically confident, GO enrichment analysis indicated that some of the FAK-RIP-enriched mRNAs were categorized as RhoGEF-related genes, Kinesin families, and endosome/lysosome membrane proteins (Supplementary Data 7), suggesting the “right mRNA for the right place” theory of mRNA transport by the FAK complex, in which the required mRNAs are specifically transported to the required locations of cellular compartments. Function-related, biased cellular distributions of RNAs and sequence-specific RNA transport have recently been reported^33,34^ and a report also suggested the sequence-specific binding of Ago2 and CCAGCC motif in mRNAs^35^, all of which support our hypothesis of specific RNA-protein interaction in the focal adhesion complex.

An important question is why the focal adhesion complex is the major and common humoral antigen in the tumor environment. Immunocytochemistry with and without permeabilization (Fig. 6a–e) revealed that, at least in in vitro settings, the focal adhesion-RNP complexes are exposed to the outside of cancer cell membranes in some occasions. Although it is technically difficult to verify this phenomenon in human cancer specimens in vivo, immunohistochemical evaluation also suggested that focal adhesion-RNP complexes might be exposed to the outer surfaces of cancer cells (Supplementary Fig. 9). On a different note, cell movement occurs through synchronized step-by-step regulation of focal adhesion maturation and lamellipodium protrusions^36,37^. Thus, it is hypothesized that cell protrusions and/or immature focal adhesion sites are physically fragile in in vivo cancer environments in which various traffic of cells and physical obstacles of the stromal matrix may interfere with cancer cell invasion. In the context of active cell movements in the fibrous cancer microenvironments, the hypothetically fragile immature focal adhesion machinery may be physically broken-down or left behind in the matrix. Those broken and naked structures of the cell adhesion machinery can be directly targeted by B cells; thus, humoral immunity against focal adhesion-RNP complexes frequently dominates in GCs. Immunohistochemical observations of FAK and the related RNP antigens in necrotic cancer microenvironments of clinical GC cases suggested that such broken and naked FAK complexes may be deposited in cancer stromal tissues (Supplementary Fig. 10), although further experiments are required to confirm this conclusion. Importantly, we observed that such focal adhesion-RNP complexes were also detected among other types of human malignancies such as lung, colorectal, and pancreatic cancers (Fig. 5 and Supplementary Figs. 5 and 6); implying that humoral immunity against the focal adhesion-RNP complexes would also dominate in various cancer environments across multiple organs. It should be underscored that the exposed focal adhesion-related RNP antigens on viable cancer cells are directly targeted by secreted antibodies. Direct biological functions of the reconstructed human antibodies, when applied to in vitro cancer cell lines, were not obvious (Supplementary Fig. 11); however, it was revealed that the amounts of exposed antigens increased when cultured under harsher conditions or with a chemotherapeutic agent (Supplementary Fig. 11). With the clinical success of lymphoma treatment via combined activations of cellular phagocytosis and antibody-dependent cellular cytotoxicity (ADCC) using anti-CD47 antibody and tumor-targeting Rituximab^38^, investigations of the therapeutic potential of anti-focal adhesion RNP antibodies against malignancies is scientifically warranted.

Most of the protein antigens identified in this study are also frequent targets of various autoimmune diseases (Table 1). To our knowledge, common biochemical and/or functional features of autoantigens in autoimmune diseases have not yet been investigated. Our findings suggest that the focal adhesion-RNP complexes revealed here to be frequent and common targets of humoral immunity in tumors are also the general triggers of autoimmune diseases. Approximately 14–25% of lung cancer patients harbor autoimmune-like disorders^39^ and a review of literatures also found statistically significant associations in the co-occurrences of various malignancies and autoimmune diseases such as SLE, PBC, MS, and MG^40^. Moreover, a case study reported that the symptoms of SLE and lupus nephritis were ameliorated by surgical resection of the tumor^41^, which clinically support the direct link between cancer-immunity and autoimmunity via a similar axis of target antigens. Our data demonstrate that these major common antigens can be focal adhesion-related RNPs. This linkage raises a biologically and clinically interesting hypothesis that proposes the existence of a disease category of “focal adhesion-related autoimmune diseases”. Precise investigations of the clinical histories of the patients (Supplementary Data 1) revealed that the GC cases from which the focal adhesion-related RNP antigens were discovered had no clinically-recognizable major autoimmune diseases^40^ prior to GC development. Therefore, emergences of the focal adhesion-related RNP antibodies were considered to be triggered by humoral reactions to cancers, not reflecting pre-existing autoimmune states.

In summary, large-scale, detailed immunogenomic analysis of tumor-infiltrating B cell repertoires in clinical GC cases identified that focal adhesion-related RNPs are major and common humoral antigens in tumors. Humoral immunity in tumor environments targets fundamental cellular compartments essential for cytoskeletal integrity and cell invasion, which sheds light on the black box of humoral immunity in tumors and hopefully point to a scientific idea to develop future immunotherapies. More focused investigation of the molecular mechanism as well as clinical applicability of the findings of this study will pave the way in the fight against cancers intractable to currently available immunotherapies.

## Methods

### Clinical samples

Frozen specimens of 102 gastric tumors and paired normal gastric mucosa that were surgically resected between 2009 and 2017 at the University of Tokyo Hospital were archived for this study under informed consent. Thirty diffuse-type GCs out of 102 GCs were also investigated in our previous report^9^. This research was approved by the Institutional Review Boards of the University of Tokyo and Tokyo Medical and Dental University. The precise clinicopathological parameters and clinical histories of all 102 patients are listed in Supplementary Data 1 and summarized in Supplementary Data 2. WES data from our previous study^10^ were integrated into the present study, and hypermutator and Epstein-Barr virus (EBV)-associated GCs were classified as in the previous study^10^. To investigate the correlation between mutation burdens, BCR entropy, and GC subtypes (Fig. 1b–d), GC cases without WES data were excluded from the analyses. For the Kaplan–Meier and multivariate regression analyses (Fig. 1e), GC cases without WES data, hypermutator GCs, and EBV-associated GCs were excluded. A log-rank test was performed to compare the overall survival of the patients.

### Helicobacter pylori (*H. pylori*) copy number analysis

*H. pylori* infection status of the patients at the time of surgery was determined using RNA-seq and quantitative-PCR (qPCR) methods. Raw sequencing reads from RNA-seq of the GC cases^10^ were aligned using STAR software in Genomon RNA analysis pipeline (https://github.com/Genomon-Project/GenomonPipeline) to the human (hg19) and the *H.pylori* genome (NC_017367). The number of paired reads in which both aligned to the *H.pylori* genome divided by those aligned to the human genome were used as the relative copy number of *H.pylori*. For the measurement of *H.pylori* copy numbers based on qPCR, extracted DNA from non-cancerous gastric tissues of the GC cases were used and qPCR was performed in our previous study^10^. Owing to the limited availabilities of genomic DNA, it was not possible to perform qPCRs for most of our GC cases.

### RNA extraction, target amplification of B cell repertoires, and next-generation sequencing (NGS)

Total RNAs of each GC and paired normal tissue were extracted from ~10 slices of serial frozen sections (10-μm thickness) using an RNeasy Mini kit (Qiagen, Germany) according to the manufacturer’s protocol. The qualification and quantification of the extracted RNA were performed on an Agilent Bioanalyzer (Agilent Technologies, CA, USA). Almost full-length variable regions, including the entire complementarity determining regions CDR1, 2, and 3, of immunoglobulin heavy and light chain genes were amplified by multiplex PCR franking the 5′ of V segments and downstream C segments following the manufacturer’s protocol (iRepertoire, AL, USA) and previous reports^9,42–44^. The PCR amplicons of the BCR/immunoglobulin repertoires were separated using an E-Gel electrophoresis system (Invitrogen, CA, USA) and purified using a QIAamp DNA Mini kit (Qiagen) according to the manufacturers’ protocols. NGS analyses utilizing a MiSeq system (Illumina, CA, USA) with 300-bp paired-end sequencing protocol were carried out to characterize the nearly full-length BCR repertoires. Low-quality sequence reads were filtered out as in the previous studies^9,44^. We used mRNA and not genomic DNA as the template for each sample for the multiplex PCR of BCR sequencing, since it is possible to obtain almost full-length and more informative repertoire data (from the V to C regions) from mRNA sequencing data; previous studies including ours have succeeded not only in evaluating precise repertoire profiles but also in reconstructing functional human IgGs, based on the mRNA sequencing data^9,45,46^. Alignment of the V(D)J recombination and CDRs of the BCR/immunoglobulin repertoire sequence reads were initially conducted by iRepertoire® (http://www.irepertoire.com/), who provided us with alignment information on the V, D, and J segments and C regions from the NGS data. Repertoire sequencing data have been deposited to the Japanese Genotype-Phenotype Archive (JGA)^47^ under accession number JGAS00000000242.

### Definition of the inverse Simpson index for the BCR repertoires

The inverse Simpson index (ISI)^48^ was used as the diversity measure for the BCR repertoire. The sequencing reads were aligned to the IMGT reference sequence using IgBlast^49^, and the clonal lineage was estimated using change-o^50,51^. The ISI value, which corresponds to Hills’s diversity index of order *q* = 2, was calculated using the ‘rarefyDiversity function of Alkazam software^50^.

### Reconstruction of tumor-infiltrating antibodies using a mammalian expression system

Tumor-specific and dominant immunoglobulin clones discovered in our repertoire sequencing of human GC tissues were reconstructed as IgG1/κ antibodies as described previously^9^. Our repertoire sequencing data lacks the 5′ short regions of IgV_H_ and IgVκ_/λ_ cDNAs; therefore, 5′ sequences inferred by the aligned V-segment information of heavy and light chains were added bioinformatically. Appropriate combinations of heavy and light chains’ pcDNA3 constructs were co-transfected into HEK293 cells and the designated IgG1/κ antibodies were secreted into and purified from culture media with the aid of ACRO Biosystems China. Even if the immunoglobulin of interest was not originally of IgG1/κ isotype in the human GC tissues, the C-region was replaced to the IgG1 isotype. The storage buffer of the antibodies was exchanged to phosphate-buffered saline (PBS) by a Zeba Desalt Spin Colum (Thermo Fisher Scientific, MA, USA).

### Cell lines

Human GC cell lines HGC27, GSU, MKN1, and KATOIII, a human immortalized fetal lung fibroblast cell line L23immo, and a human pancreatic carcinoma cell line Panc-1 were obtained from RIKEN BioResource Research Center (BRC) (Japan). A human GC cell line NUGC-3, a human monocytic leukemia cell line THP-1, a human promyelocytic leukemia cell line HL60, and a human lung adenocarcinoma cell line A549 were obtained from Japanese Collection of Research Bioresources (JCRB) Cell Bank (Japan). Cells were cultured in RPMI1640 medium (#189-02025, FUJIFILM Wako Pure Chemical Corporation, Japan) supplemented with 10% fetal bovine serum (FBS) (#172012, Sigma-Aldrich) (with exceptions of KATOIII and HL60 that were cultured with 20% FBS), 2 mM L-glutamine (#073-05391, FUJIFILM Wako Pure Chemical Corporation), Penicillin/Streptomycin (#168-23191, FUJIFILM Wako Pure Chemical Corporation), and 1 mM sodium pyruvate (#11360-070, Thermo Fisher Scientific). Negativity for Mycoplasma infection has been regularly confirmed using TaKaRa PCR Mycoplasma Detection Set (TaKaRa Bio, Japan).

### Immunoprecipitation and mass spectrometry analysis

For the global screening of protein antigens of the reconstructed antibodies, immuno-precipitations followed by mass spectrometry analyses were performed. Total cellular proteins were extracted from confluent 10-cm dishes of human GC cell lines (HGC27, GSU, and NUGC3), a human monocytic leukemia cell line (THP-1), a human promyelocytic leukemia cell line (HL60), and a human immortalized fetal lung fibroblast cell line (L23immo), using a lysis buffer containing 50 mM Tris (Sigma-Aldrich, MO, USA), 150 mM NaCl (FUJIFILM Wako Pure Chemical Corporation), and 1% Triton-X100 (Sigma-Aldrich) with a proteinase inhibitor cocktail (Roche Diagnostics, Switzerland). The extracted cell lysates were then incubated with 2.0 μg reconstructed human antibodies to precipitate their antigens, if any, for an overnight. The antibody-protein complex was magnetically purified by protein G beads (Bio-Rad Laboratories, CA, USA) as instructed. Protein antigens specific to the reconstructed human antibodies were separated by 10% SDS-PAGE and the protein bands were visualized by Coomassie Brilliant Blue (Bio-Rad Laboratories). Diluted antibodies without protein lysate were also run by electrophoreses as negative controls. Parallel immunoprecipitation experiments using unrelated human antibodies were also performed as negative controls. Any specific protein bands of the reconstructed human antibodies found in the SDS-PAGE gels were analyzed by mass-spectrometry QTRAP (Applied Biosystems, CA, USA); then, the protein antigens were characterized. For antibodies for which no specific protein bands were discovered by the immunoprecipitation protocol described above, we also tried experiments using more stringent lysis buffer; e.g., RIPA buffer, confirming that no specific protein bands could be found for those antibodies. For the presented CBB stained SDS gels in Fig. 2, uncropped images are shown in Supplementary Fig. 12.

Co-immunoprecipitations (IPs) using anti-FAK antibody (AHO0502, Thermo Fisher Scientific) used the MKN1 human GC cell line since it was considered that MKN1 cell line expresses higher levels of FAK-associated proteins except for GRB7 by our prior observation. The GSU human GC cell line was used for the immunoprecipitation using anti-GRB7 antibody (PA5-79323, Thermo Fisher Scientific). To validate the co-IP of MKN1 and GSU cells, A549 cell and GRB7-overexpressing HEK293 cells were also used. The experimental protocol for immunoprecipitation was the same as the one described above except for the application of 1.0 μg of antibodies in each experiment. Polyclonal rabbit IgG (ab37415, Abcam, UK) (1.0 μg each) were used as negative controls.

### Antigen screening by protein array

For orphan antibodies for which specific antigens were not successfully identified in the above-mentioned experiments, we performed protein array assays (HuProt^TM^ Human Proteome Microarray v4.0) (CDI Laboratories, PR, USA) to explore any candidate protein antigens. One-microgram mixtures of the orphan reconstructed human antibodies were applied to the experiments with the aid of CDI Laboratories. The top-ranked candidate antigens were evaluated by independent immunoprecipitation-immunoblot experiments and one protein, GRB7, was confirmed to be a specific antigen of an orphan antibody.

### Immunoblotting

For the antigen screening, immunoblots were also performed of the immunoprecipitated proteins to confirm the results of mass spectrometry and protein array. The human GC cell lines HGC27, GSU, NUGC3, MKN1, KATOIII, and L23immo grown to confluency in 10-cm dishes were first used for immuno-precipitation by the reconstructed human antibodies. Then, immunoblots using the following antibodies were performed: anti-FLNA (HPA002925, Sigma-Aldrich), anti-PABP1 (#4992, Cell Signaling Technology, MA, USA), anti-DLAT (GTX109766, GeneTex, CA, USA), anti-LMNA (ab108595, Abcam), anti-EML3 (PA5-71024, Thermo Fisher Scientific), and anti-GRB7 (PA5-79323, Thermo Fisher Scientific). Tris-buffered saline with 0.5% Tween-20 supplemented with non-fat dry milk (#190-12865, FUJIFILM Wako Pure Chemical Corporation) was used as a protein blocking solution for immunoblots.

For the co-immunoprecipitation experiments using anti-FAK (AHO0502, Thermo Fisher Scientific) and anti-GRB7 (PA5-79323, Thermo Fisher Scientific) antibodies, immunoblots were performed using anti-FLNA (HPA002925, Sigma-Aldrich), anti-EZR (#07-130, Merck Millipore, MA, USA), anti-HSP90 (#07-2174, Merck Millipore), anti-PABP1 (ab21060, Abcam), anti-FAK (AHO0502, Thermo Fisher Scientific), and anti-GRB7 (ab109618, Abcam) antibodies. All immunoblot signals were recorded using a ChemiDoc^TM^ Touch Imaging System (Bio-Rad Laboratories) according to its manual.

Uncropped immunoblot images of the figures are shown in Supplementary Fig. 12.

### Gene ontology (GO) enrichment analysis

GO enrichment analyses were conducted for selected gene sets of interest by the Enrichr platform (https://amp.pharm.mssm.edu/Enrichr/). For the nine identified protein antigens of the tumor-specific human immunoglobulins, the GO Cellular Component database was used to enumerate the enrichment scores, while the GO Molecular Function database was used for five selected genes of the focal adhesion complex proteins. All databases used were as of May 31, 2019.

### Immunocytochemistry, immunofluorescent, and immunohistochemistry staining

Dual-color Immunocytochemistry of the MKN1 and GSU human GC cell lines was performed as follows. Cells sparsely grown on an eight-well Chamber Slide System (Thermo Fisher Scientific) were fixed with 4% paraformaldehyde (PFA) for 10 min and, if needed, permeabilized with 0.05% Triton-X-100 for 5 min; the non-specific protein reactions were then blocked with 2% bovine serum albumin (BSA) (A1470, Sigma-Aldrich)/PBS for 30 min. Mixtures of primary rabbit and mouse antibodies were applied to the slide chambers and incubated for 1 h at room temperature. The antibodies used were as follows: rabbit anti-EZR (#07-130, Merck Millipore), anti-FLNA (HPA002925, Sigma-Aldrich), anti-PABP1 (ab21060, abcam), anti-DLAT (GTX109766, GeneTex), anti-LMNA (ab108595, Abcam), anti-EML3 (PA5-71024, Thermo Fisher Scientific), anti-GRB7 (PA5-79323, Thermo Fisher Scientific), and anti-RPS6 (#2217S, Cell Signaling Technologies) antibodies, as well as a mouse anti-FAK (#05-537, Merck Millipore) antibody. All primary antibodies were diluted at 1:100 in 2% BSA/PBS. Goat anti-rabbit Alexa-488 and anti-mouse Alexa-568 antibodies (at 1:100 dilutions) (A-11008 (Thermo Fisher Scientific) and ab175701 (Abcam), respectively) were mixed and used as secondary antibodies. The secondary antibodies were incubated for 1 h at room temperature.

We also performed dual-color immunofluorescent analysis of clinical GC tissues using the same primary and fluorescent secondary antibodies mentioned above, and we additionally used anti-Alpha II-spectrin (A301-249A, Bethyl Laboratories). Formalin-fixed and paraffin-embedded GC specimens were deparaffinized by immersion in xylene (#241-00091, FUJIFILM Wako Pure Chemical Corporation) for 10 min at room temperature; antigen retrieval was then performed by autoclave using a citric acid buffer (pH = 6.0) (ab64214, Abcam) for 5 min at 121 °C. Blocking of non-specific reactions was performed by 2.0% BSA (Sigma-Aldrich)/PBS. Mixed mouse and rabbit primary antibodies were applied to the slides and then incubated for an overnight at 4 °C. After washing three times with PBS, mixed fluorescent secondary antibodies were applied for 1 hr at room temperature. Immunohistochemistry on clinical GC specimens was also performed using anti-CD4 (ab133616, abcam), CD20 (ab78237, abcam), CD68 (ab955, abcam), CD138 (#36-2900, Thermo Fisher Scientific) antibodies. Histopathological GC specimens were deparaffinized and antigens were retrieved as described above; then, the slides were immersed in 0.3% H_2_O_2_ (#081-04215, FUJIFILM Wako Pure Chemical Corporation) / methanol (#137-01823, FUJIFILM Wako Pure Chemical Corporation) for 10 min at room temperature to remove endogenous peroxidase. Blocking of non-specific reactions was performed by incubating the slides with 2.0% BSA/PBS. Primary antibodies were incubated on the slides for an overnight at 4 °C. After washing for 3 times by PBS, the antigen-antibody complexes on the slides were visualized by using Histostar (#8460, MBL, Japan) and DAB Substrate Solution (#8469, MBL). Finally, cell nuclei were stained by hematoxylin (Sakura Finetech, Japan, # 8650).

For both immunocytochemistry and immunofluorescent examinations, cell nuclei were stained with Hoechst 33342 (#4082S, Cell Signaling Technologies) and the slides were mounted with Prolong Gold anti-fade reagent (#P36935, Invitrogen). The fluorescence signals were evaluated using a fluorescent microscope Leica AF6000 system (Leica, Germany) according to the manufacturer’s manual.

### Single-strand RNA staining and Cy5-tagged oligo-dT hybridization

To visualize the distribution of cellular RNA, we performed single-strand RNA staining and oligo dT hybridization in combination with immunocytochemistry. The human GC cell line MKN1 grown sparsely in an 8-well Camber Slide System (Thermo Fisher Scientific) was fixed with 4% PFA for 10 min and permeabilized with 0.05% Triton X-100/PBS for 5 min. Non-specific reactions were then blocked with 2% BSA (Sigma-Aldrich)/PBS for 30 min. Cellstain® Acridine Orange (Dojindo, Japan) or Cy5-conjugated oligo dT (25mer) probe (synthesized by eurofins Genomics, Japan), each of which could detect single-stranded RNA or polyadenylated mRNA, respectively, were incubated on the slides overnight at 4 °C. Anti-FAK antibody (#05-537, Merck Millipore) was also incubated on the slides in combination with the probes. Cell nuclei were visualized by Hoechst 33342 (Cell Signaling Technologies).

### RNA immunoprecipitation (RIP) and RNA-seq

MKN1 human GC cells grown to confluency in eight 15-cm dishes were used for RNA immunoprecipitation (RIP). The cellular protein-RNA complexes were cross-linked by the drop-wise addition of 4% PFA to the culture medium to a final concentration of 0.75% and incubated for 10 min. The cross-link reaction was stopped by the addition of glycine (G8898, Sigma-Aldrich) to a final concentration of 125 mM and the cells were further incubated for 5 min. The cells were rinsed twice with 10 mL ice-cold PBS and then thoroughly scraped with 5 mL of ice-cold PBS using a cell scraper (MS-93300, Sumitomo Bakelite, Japan). The fixed cells were lysed using a buffer containing 50 mM Tris (Sigma-Aldrich), 150 mM NaCl (FUJIFILM Wako Pure Chemical Corporation), and 0.1% Triton-X100 (Sigma-Aldrich) with proteinase inhibitors (#11873580001, Sigma-Aldrich) and RNase inhibitor (#2313A, Takara Bio, Japan). Debris was excluded by centrifuging at 14,000 rpm for 5 min at 4 °C. The following procedures were carried out with buffer containing RNase inhibitor. To pre-clean non-specific reactions, the initial cell lysate was incubated with 8.0 ug control polyclonal rabbit IgG (ab37415, Abcam) for 1 h at 4 °C; non-specific IgG-protein complexes were removed using protein G beads (#1614023, Bio-Rad Laboratories). The cell lysate was separated into two tubes, each of which was incubated with 1.0 μg anti-FAK antibody (AHO0502, Thermo Fisher Scientific) or control polyclonal rabbit IgG (ab37415, Abcam) overnight at 4 °C. The FAK-RNA complex was immunoprecipitated by protein G beads (#1614023, Bio-Rad Laboratories) and the protein-RNA crosslinks were reversed by adding 250 mM NaCl (FUJIFILM Wako Pure Chemical Corporation) and 300 ug/mL proteinase K (Sigma-Aldrich) and incubated at 65 °C for 1 h with shaking. The precipitated RNA was purified using an RNeasy mini kit (Qiagen).

Purified RNA was qualified and quantified using an Agilent Bioanalyzer (Agilent) and rRNA was eliminated using an NEBNext® rRNA Depletion kit (New England Biolabs, MA, USA); a SMART-Seq® Stranded Kit (#634442, Takara Bio) was then used to construct an RNA-seq library according to the manufacturer’s protocol. The RNA-seq library was further processed to be applied to an Ion PGM system (Life Technologies, CA, USA) using an Ion Plus Fragment Library Kit (Thermo Fisher Scientific) as instructed.

Sequencing reads from an Ion PGM system were trimmed using Trimmomatic^52^ (options: LEADING:20 TRAILING:20 MINLEN:30), illumina’s adapter sequences and poly-A tails were trimmed by Cutadapt software^53^, and sequence reads mapped to human rDNA sequences (GenBank ID: U13369.1 and X12811.1) were removed using bwa^54^. The remaining sequencing reads were inputted to salmon with GENCODE v30 referencing all the human transcriptome to quantify the transcript-level abundance. Using the tximport package^55^, these transcript-level abundances were integrated to gene-level abundances for the subsequent analysis. The FAK-RIP data can be found in Supplemental Data 5, 6, and 7.

### De novo motif identification of untranslated region (UTR) regulatory elements in precipitated RNA

The 5′ and 3′ UTR sequences of genes were retrieved using TxDB^56^. When multiple UTRs existed in a single gene, the longest UTR sequence was selected as the representative UTR. We defined two groups of mRNAs containing mRNAs enriched in FAK-RIP samples (5-fold higher than the control sequencing of cellular total-mRNA) and containing less-abundant mRNAs in FAK-RIP samples (5-fold less than the control), respectively. Then, the UTR sequences in each group were entered into MEME suite^57^ in differential enrichment mode with the ‘scan given strand only’ option to extract motifs enriched within the UTR sequences of the FAK-RIP-enriched genes.

### Flow cytometry

Flow cytometry analyses were performed for various human cancer cell lines, including GCU and A549. Cells were cultured under conditions as indicated in Supplementary Fig. 11; then, cells were incubated with our reconstructed immunoglobulins. Alexa-488 conjugated goat anti-human IgG antibody (A11013, invitrogen) was used as a secondary antibody. FITC signals were detected by NovoCyte (ACEA Biosciences, CA, USA) and analyzed by NovoExpress (ACEA Biosciences).

### MTT assay

MTT assays were performed using Cell Proliferation Kit I (MTT) (Roche Diagnostics, Switzerland) according to the manufacture’s protocol. Cells were cultured in 96 well plates with either control IgG (Human IgG (Normal)) (Invitrogen) or our reconstructed Abs (20 μg/ml, when not otherwise specified). Starting cell numbers were as follows; KatoIII: 1.0 × 10^4^, GSU: 8.0 × 10^3^, HGC27: 5.0 × 10^3^, NUGC3: 6.0 × 10^3^, MKN1: 5.0 × 10^3^. Four days later, 10 μl MTT Labeling Reagent was added into the culture medium for 4 h, then 100 μl Solubilization Solution was added and incubated for an overnight. Absorbance at 550 nm was measured for each well and the control blank well value was subtracted. Experiments were done at least in tetra-plicate.

### Statistics and reproducibility

Mann–Whitney *U* test and *t*-test were used both by two-sided to test the statistical significance of differences between groups. Kaplan–Meier analysis, log-rank test, and multivariate Cox regression model were performed for survival analyses. E-values in the motif enrichment analysis were calculated in MEME suite platform (http://meme-suite.org/). In the GO enrichment analyses, adjusted *p* values for each category were calculated by Enrichr platform (https://amp.pharm.mssm.edu/Enrichr/). Immunoprecipitation experiments were performed at least twice, MTT assays were performed at least in tetraplicates for at least twice, and FAK-RIP experiment was performed using biological duplicates of RIP samples, to confirm reproducibility. Evaluations of immunocytochemistry and immunofluorescent staining were performed for multiple of cells with visible formations of focal adhesions and for multiple microscopic fields, respectively, to confirm reproducibility.

### Reporting summary

Further information on research design is available in the Nature Research Reporting Summary linked to this article.

## Supplementary information

Supplementary Information

Description of Additional Supplementary Files

Supplementary Data 1-7

Reporting Summary

## Data Availability

The BCR repertoire sequencing dataset generated during the current study is available in the Japanese Genotype-phenotype Archive (JGA)^47^ under an accession number JGAS00000000242. The RNA-seq dataset of the FAK-RIP experiment in this study is available in DDBJ Sequence Read Archive (DRA) under an accession number DRA010767. Other datasets generated during the current study are available from the corresponding authors on reasonable request.
