# Supplementary material for: Focal adhesion ribonucleoprotein complex proteins are major humoral cancer antigens and targets in autoimmune diseases
Source: Commun Biol. 2020 Oct 16;3:588. doi: 10.1038/s42003-020-01305-5 (PMC7567837; doi:10.1038/s42003-020-01305-5)
Supplement: Supplementary file 1 — Supplementary Information [file 42003_2020_1305_MOESM1_ESM.pdf]

Supplementary Fig. 1

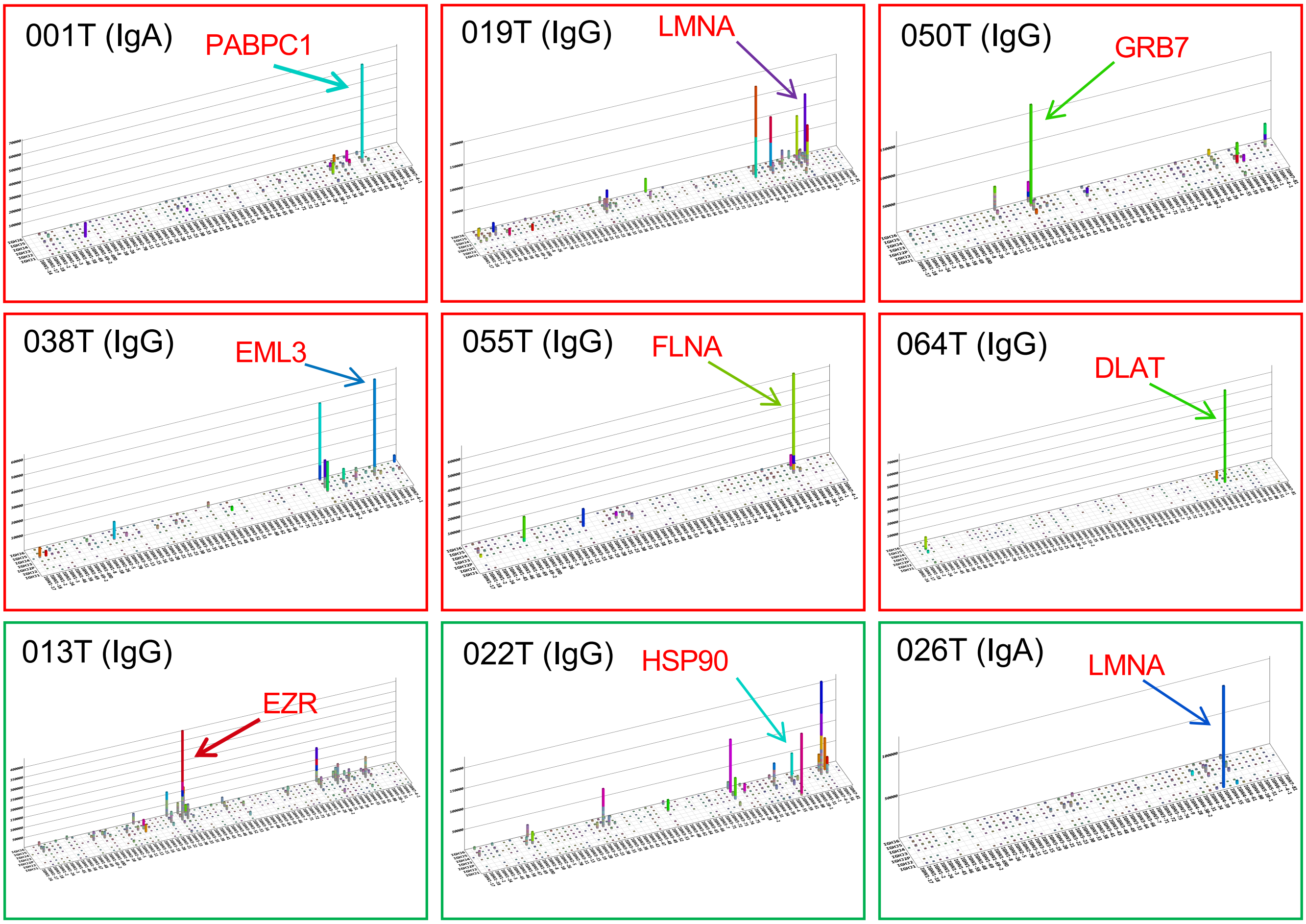

Supplementary Fig. 1: Three-dimensional plots of immunoglobulin repertoire profiles of tumor-infiltrating B cells in GC microenvironments. The numbers on the left-upper corner represent the GC case IDs. Only cases for which specific protein antigens were identified are shown. The x and y-axes represent the V and J segments of the Immunoglobulin variable region heavy chain (IgVH) genes, respectively, while the z-axis represents the numbers of NGS reads. The bars are multi-colored according to the CDR3 clonotype groups to highlight the dominant CDR3s in each case. The red squares indicate immunoglobulins for which the protein antigens were newly identified in this study. The green squares indicate immunoglobulins whose protein antigens were identified in our previous study (Katoh et al., 2017).

Supplementary Fig. 2

Overview of the identification of protein antigens for the tumor-specific dominant immunoglobulin clones

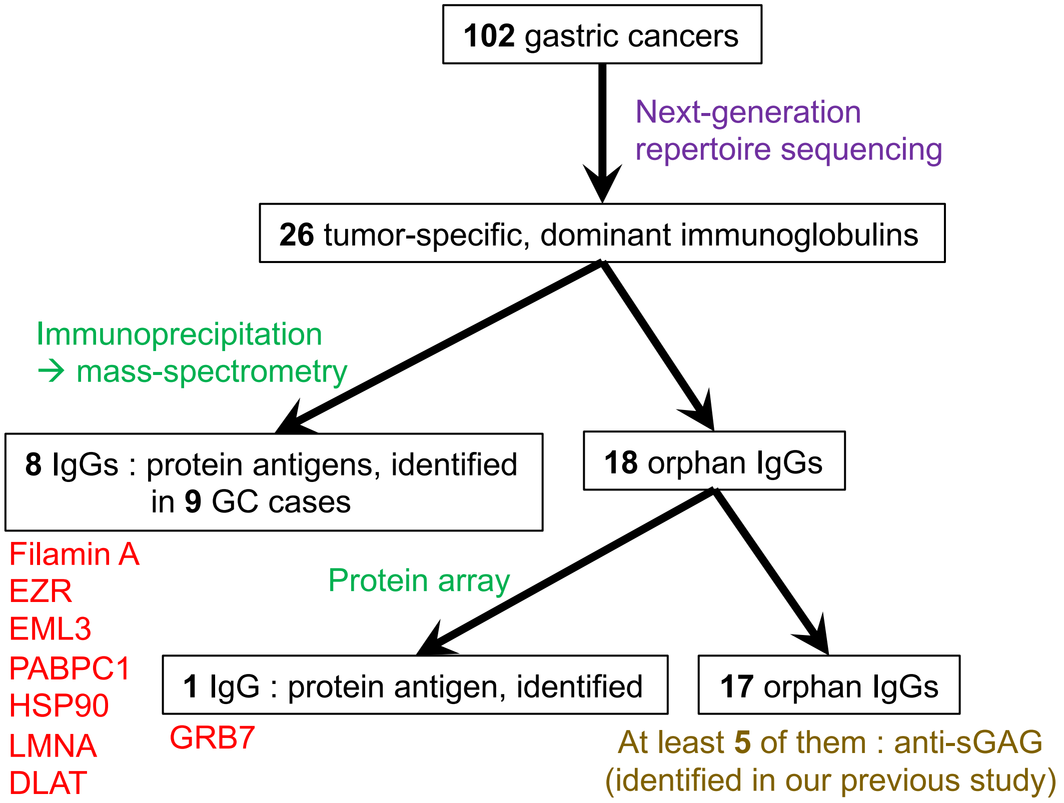

Supplementary Fig. 2: An overview of the flow of our screening for the identification of protein antigens corresponding to tumor-specific dominant immunoglobulin clones.

Supplementary Fig. 3

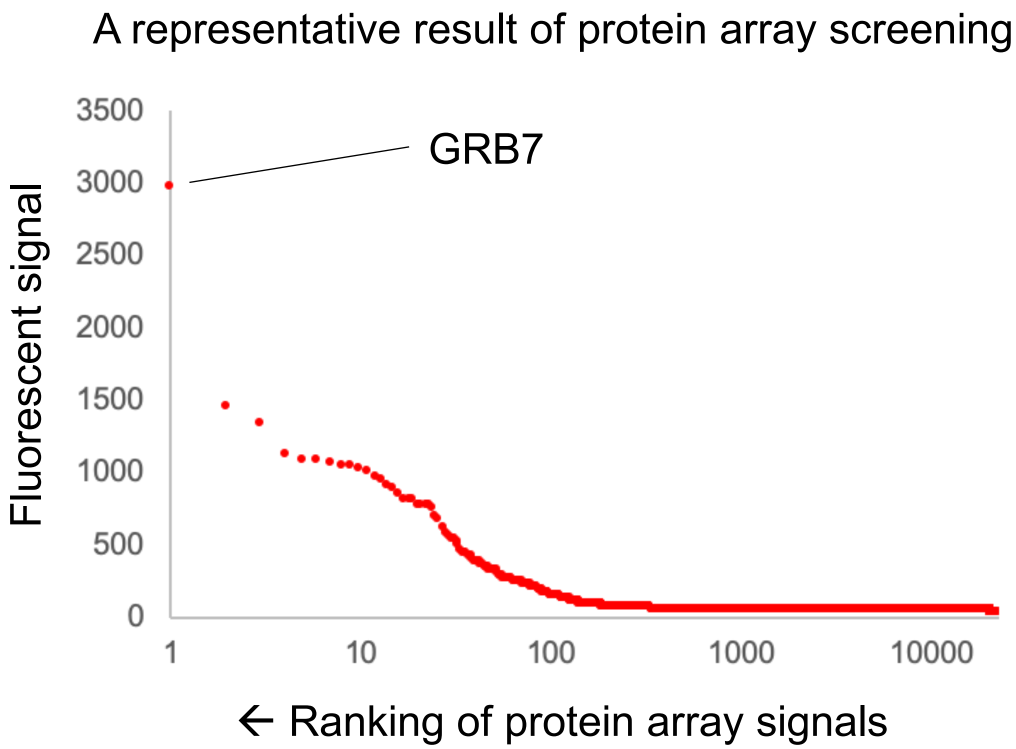

Supplementary Fig. 3: A representative result of protein antigen screening for the reconstructed human immunoglobulins using a protein array (HuProt®) as described in Methods. Orphan immunoglobulins for which the corresponding protein antigens had not been identified were subjected to the protein array experiments. One protein antigen, GRB7, was successfully identified in this screen. Each red dot represents the fluorescent signal of a protein spot on the array. The x and y-axes represent the ranks and values of the fluorescent signals, respectively. This result was confirmed by independent immunoprecipitation experiment, as shown in Fig. 2.

Supplementary Fig. 4

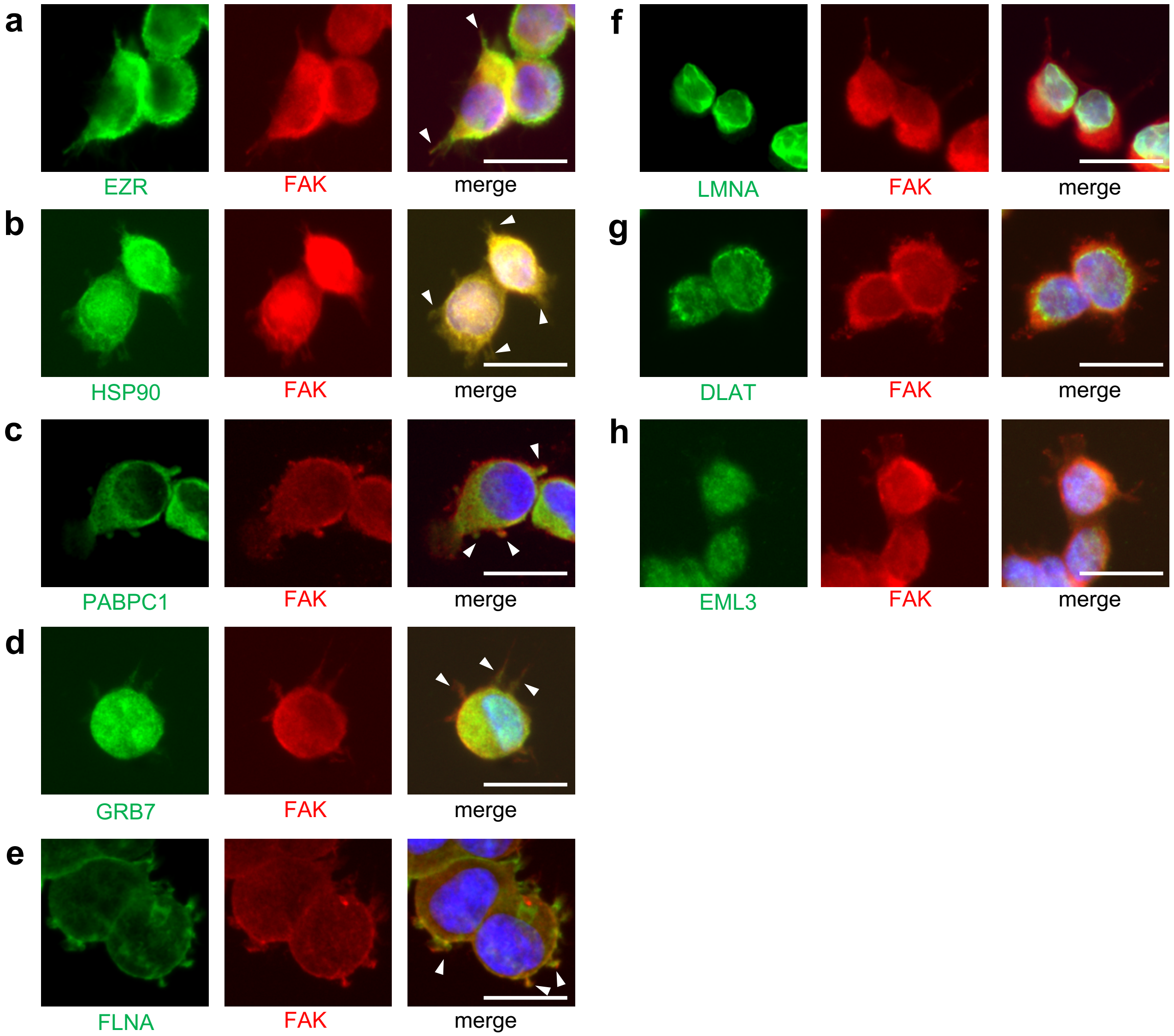

Supplementary Fig. 4: Fluorescent immunocytochemistry of the identified protein antigens along with FAK. **a-h** Fluorescent dual-immunocytochemistry using antibodies for the identified protein antigens (green) along with FAK (red) for a human gastric cancer cell line GSU. Blue color represents nuclear staining of Hoechst. White bars indicate 20μm. White arrow heads; representative hot spots of colocalization between protein antigens and focal adhesions. **a-e** Similar colocalizations were reproducibly observed in all analyzed cells with formations of visible focal adhesions (**a-e**).

Supplementary Fig. 5

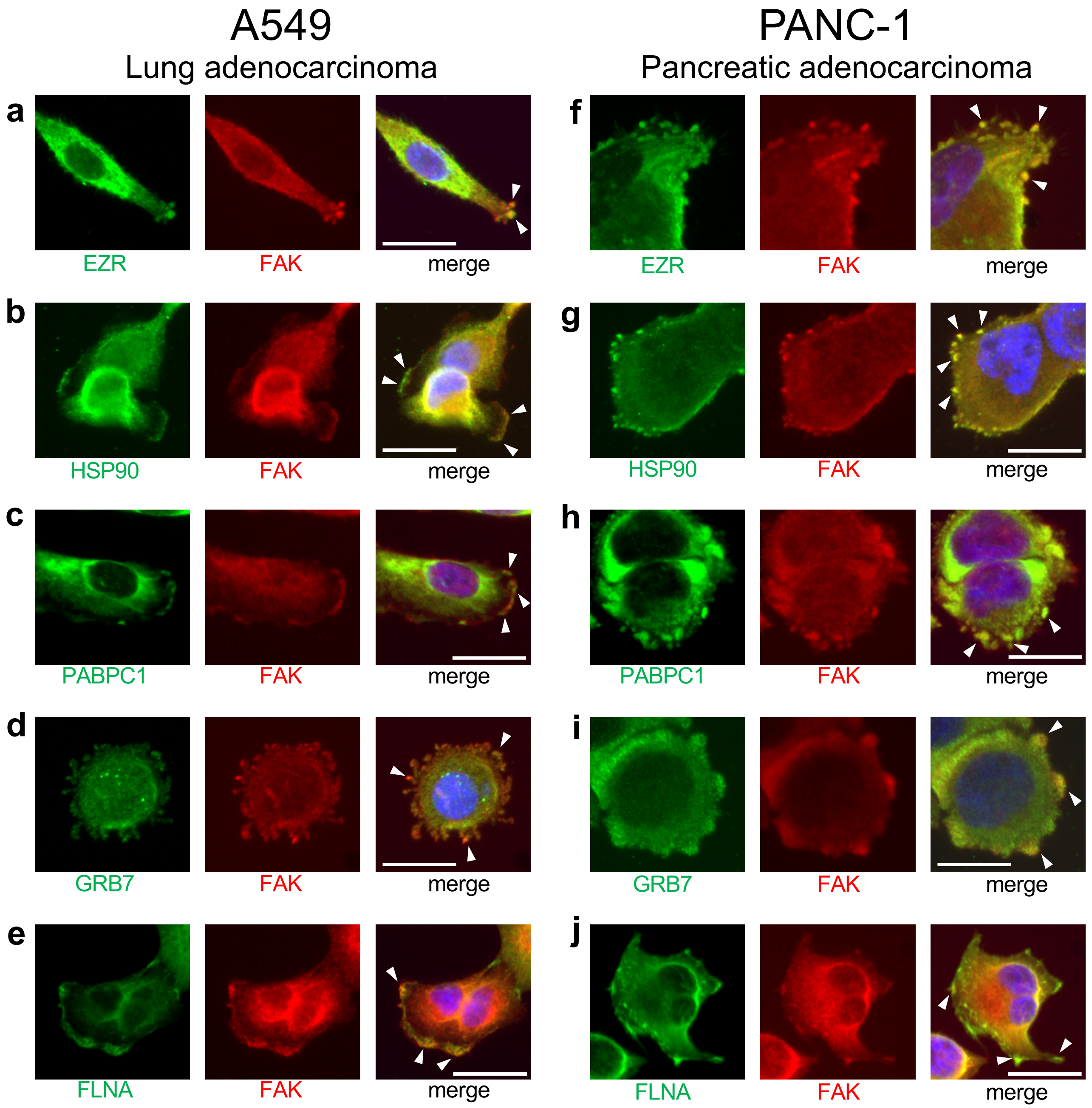

Supplementary Fig. 5: Fluorescent immunocytochemistry of the identified protein antigens along with FAK. **a-j** Fluorescent dual-immunocytochemistry using antibodies for the identified protein antigens (green) along with FAK (red) for a human lung adenocarcinoma cell line A549 (**a-e**) and a human pancreatic cancer cell line PANC-1 (**f-j**). Blue color represents nuclear staining of Hoechst. White bars indicate 20μm. White arrow heads; representative hot spots of colocalization between protein antigens and focal adhesions. Similar colocalizations were reproducibly observed in all analyzed cells with formations of visible focal adhesions.

Supplementary Fig. 6

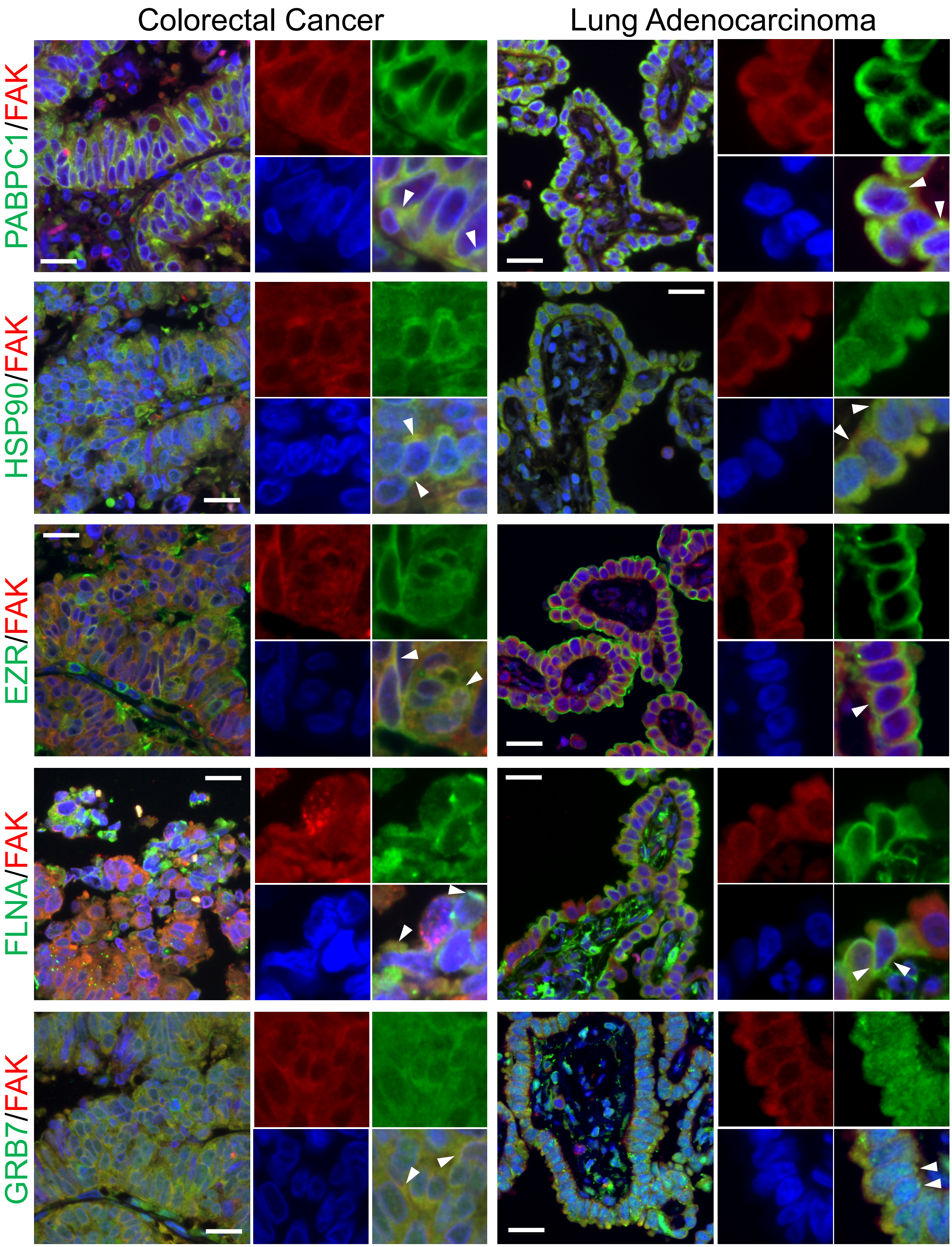

Supplementary Fig. 6: Co-localizations of the identified protein antigens with focal adhesions in clinical specimens of colorectal carcinoma and lung adenocarcinoma. Fluorescent immunohistochemistry of the identified protein antigens (green) along with FAK (red) was performed as in Fig. 4. The white arrowheads indicate representative colocalization of protein antigens and focal adhesions. White bars indicate 20μm. Clear colocalizations were reproducibly observed in multiple of cancer cells in at least five randomly selected microscopic fields in these clinical samples.

Supplementary Fig. 7

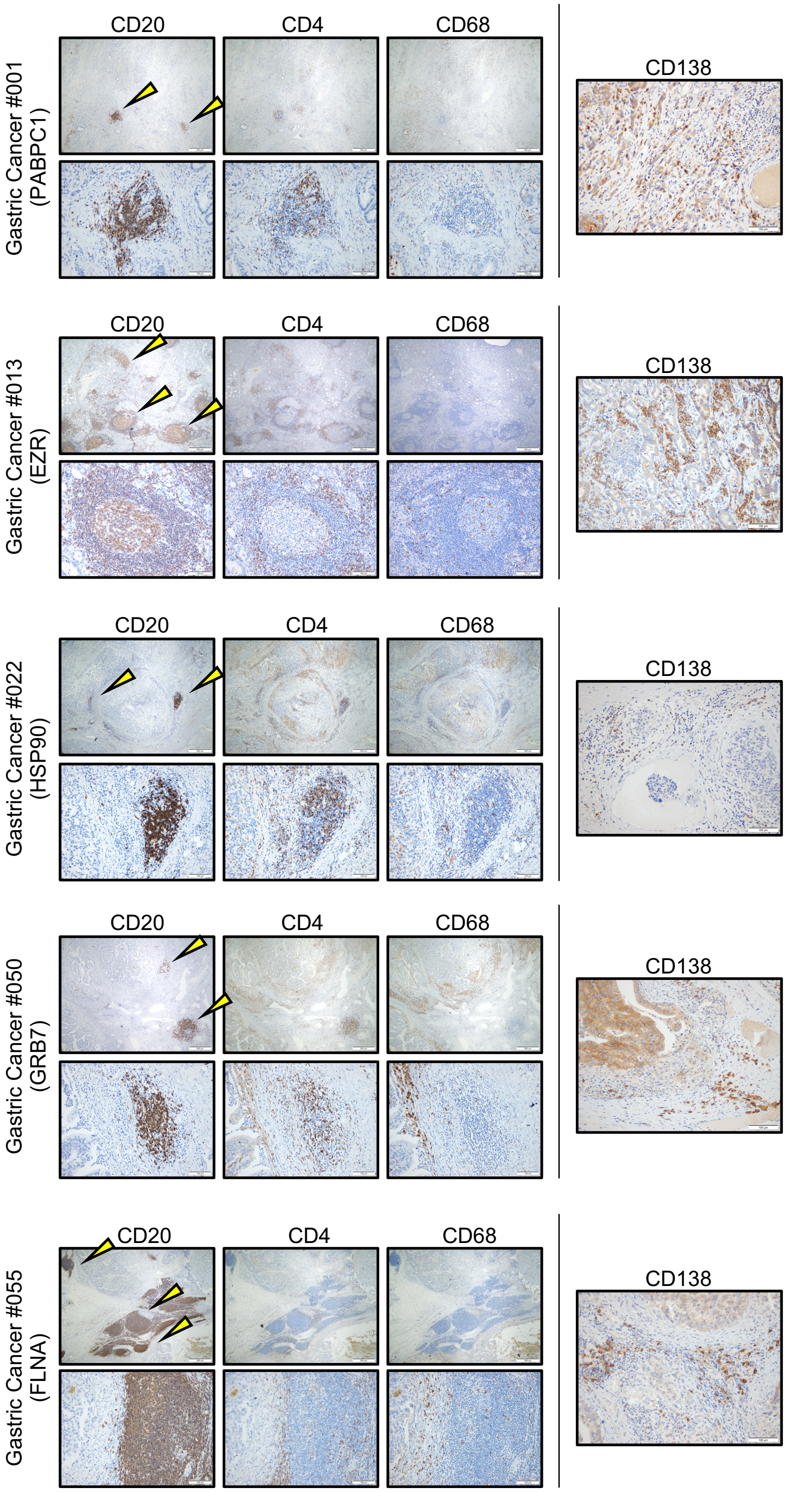

Supplementary Fig. 7: Representative immunohistochemical evaluations of tumor-infiltrating immune cells in GC cases for which the anti-FAK-related RNP antibodies were identified. Formations of multiple of tertiary lymphoid structures with germinal centers and/or lymphoid follicles, as represented by yellow arrow heads, suggested active humoral immunity in these tumor environments (left panels). CD20, CD4, and CD68 indicate B cells, $CD4^{+}$ T cells, and macrophages, respectively. Furthermore, $CD138^{+}$ plasma cell infiltration into GC microenvironments was robustly observed in all of these GC cases (right panels). Scale bars in the upper and lower panels indicate 500 μm and 100 μm, respectively.

Supplementary Fig. 8

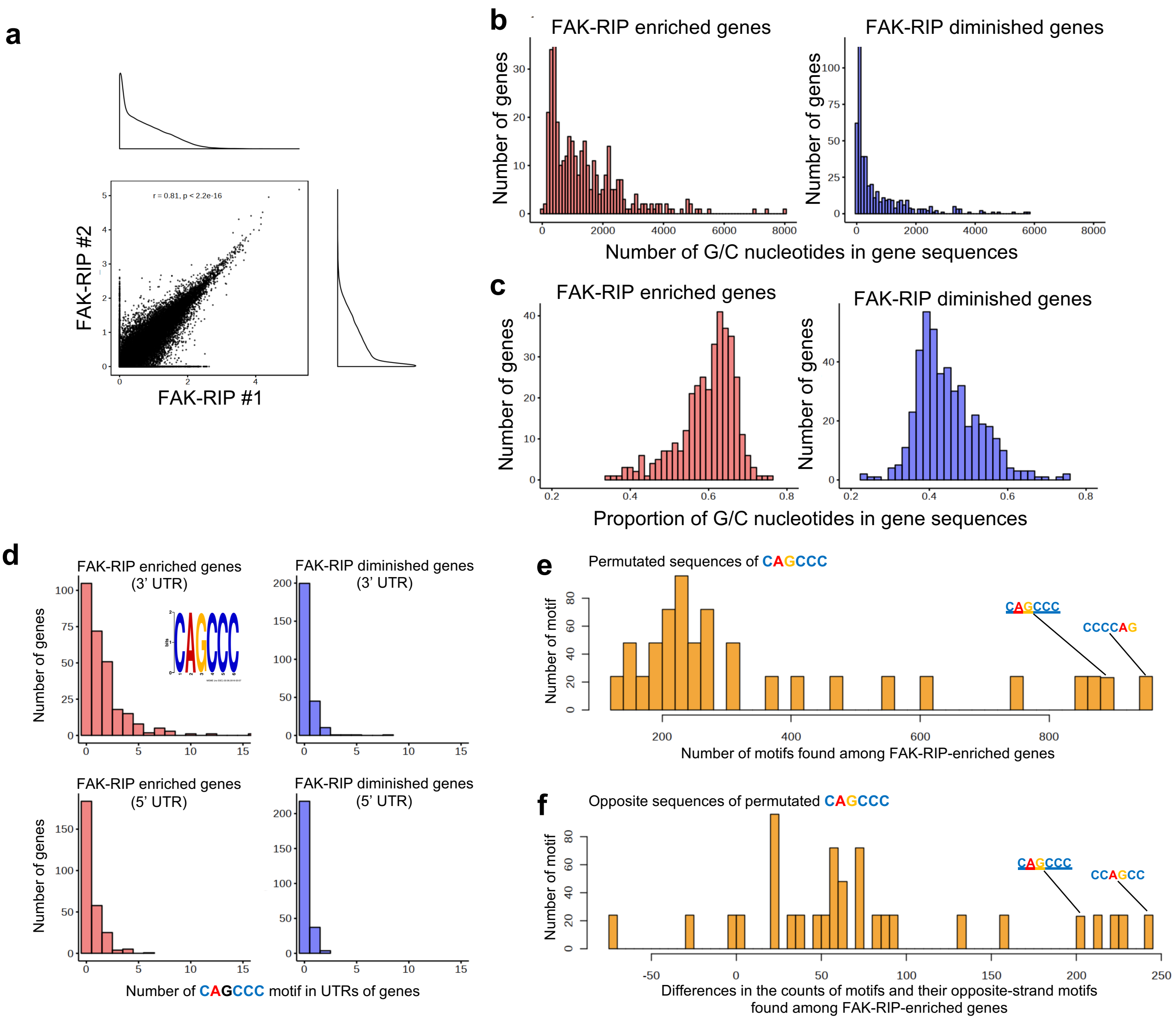

Supplementary Fig. 8: Motif enrichment analysis of the FAK complex-bound mRNAs/transcripts. **a** Reproducibility of the two biological replicates of FAK-RIP experiments. Each dot represents NGS sequence reads for every transcript. Only genes that were reproducibly enriched (at least five-fold) compared to the control were included in the following analysis (Fig. 5g). **b-c** Numbers and proportions of G/C contents were apparently higher among FAK-RIP-enriched transcripts. **d** One of the top-ranked motifs, CAGCCC (as shown in Fig. 5h), was substantially enriched not only in the comparisons within 3' and 5' UTRs (upper and lower panels, respectively) but also in the comparison of the entire gene sequences (Fig. 5i). **e-f** Enrichment of the CAGCCC motif was not fully attributable to the G/C-rich nature of the FAK-RIP-enriched genes since permutated sequences (**e**) and their opposite-strand sequences (**f**) of CAGCCC did not comparably dominate among the FAK-RIP-enriched genes.

As shown in Fig. 5h, the G/C-enriched sequences appear to stand out among the FAK-RIP-enriched genes, which might reflect non-specific binding between the FAK complex and G/C-rich RNAs. However, as shown in Supplementary Figs. 8e-f above, the FAK-RIP-enriched CAGCCC motif and its similar sequences were observed specifically and at high frequency among FAK-RIP-enriched genes. Together, these findings suggest that the FAK complex plays an important role in the sequence-specific mRNA transport system.

Supplementary Fig. 9

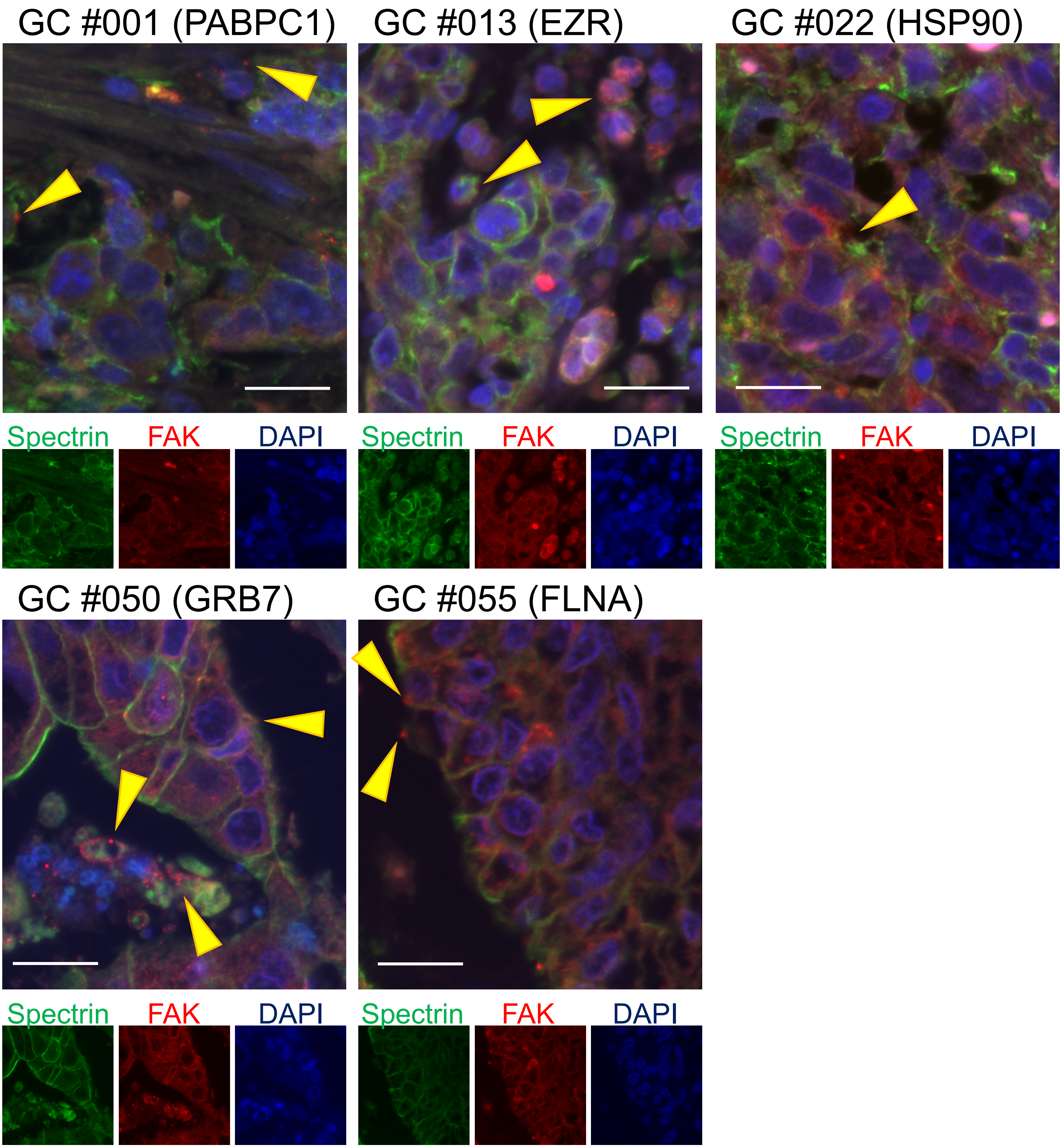

Supplementary Fig. 9: Possible exposure of FAKs of tumor cells in clinical samples. We evaluated whether the FAKs are exposed to the outside of cells in clinical GC samples by immunohistochemistry, although such an evaluation might be technically challenging. Spectrin (green) is known to line the inner side of cellular membranes and was considered to indicate the edges of cellular membranes. Yellow arrowheads indicate FAKs (red) that are possibly exposed to the outside of the cellular membrane. White bars indicate 20μm. In all clinical GC cases from which the FAK-related RNP antigens were identified, such possible exposures of FAKs were detected, albeit rarely. In combination with the data from *in vitro* experiments (Fig. 6), it can be concluded that FAK, at least in some instances, is topologically exposed to the outside of tumor cells.

Supplementary Fig. 10

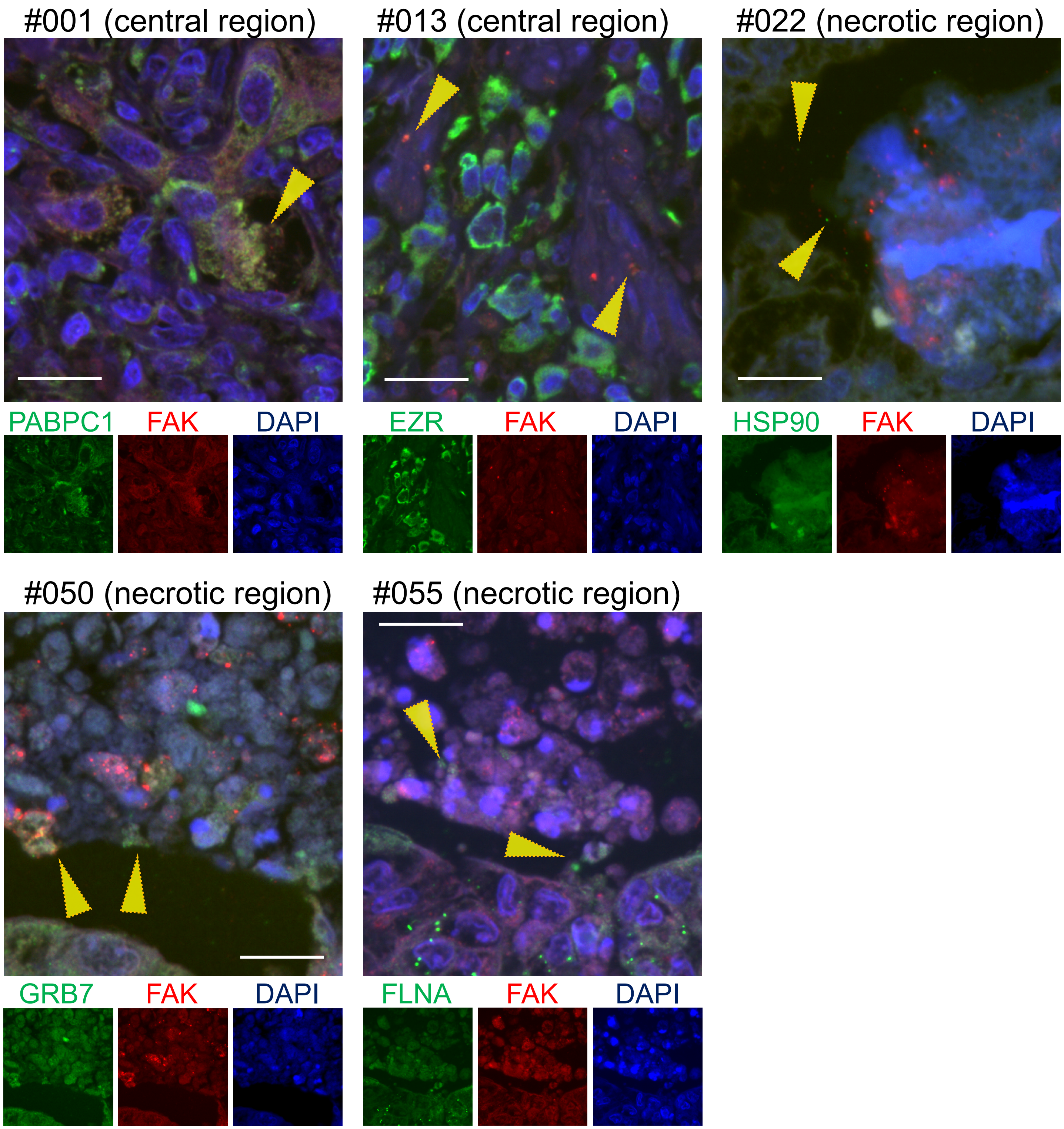

Supplementary Fig. 10: Possible depositions of naked FAK complexes in necrotic and stromal tissues of clinical samples. We evaluated whether the FAK complex is deposited in tumor environments in clinical GC samples by immunohistochemistry, although such an evaluation might be technically challenging. Necrotic regions of the GC cases examined in Fig. 4 were re-evaluated and possible small depositions of the FAK-related RNP antigens identified in this study (green), either complexed with or independent of the FAKs (red), were frequently observed in all clinical GC samples from which the RNP antigens themselves were identified. White bars indicate 20μm. Although it is technically challenging to obtain robust conclusions, it can be hypothesized that FAK-related RNP complexes might be deposited in necrotic and/or stromal tumor environments in clinical samples.

Supplementary Fig. 11

a

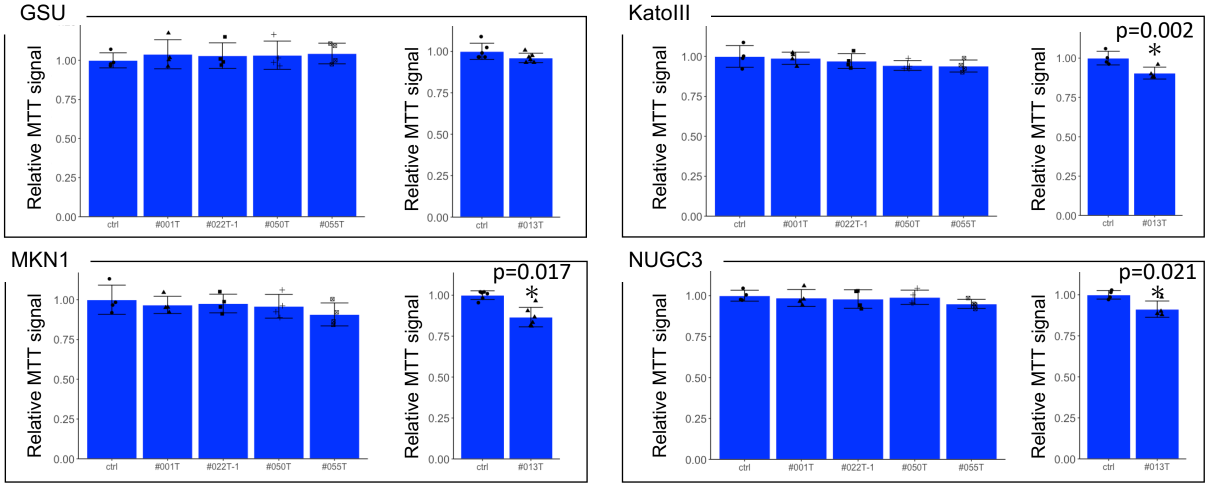

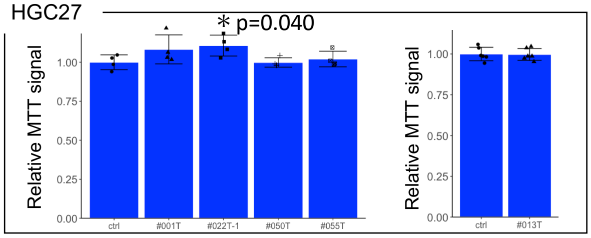

Antibody	#001T	#022T-1	#050T	#055T	#013T
antigen	PABPC1	HSP90	GRB7	FLNA	EZR

b

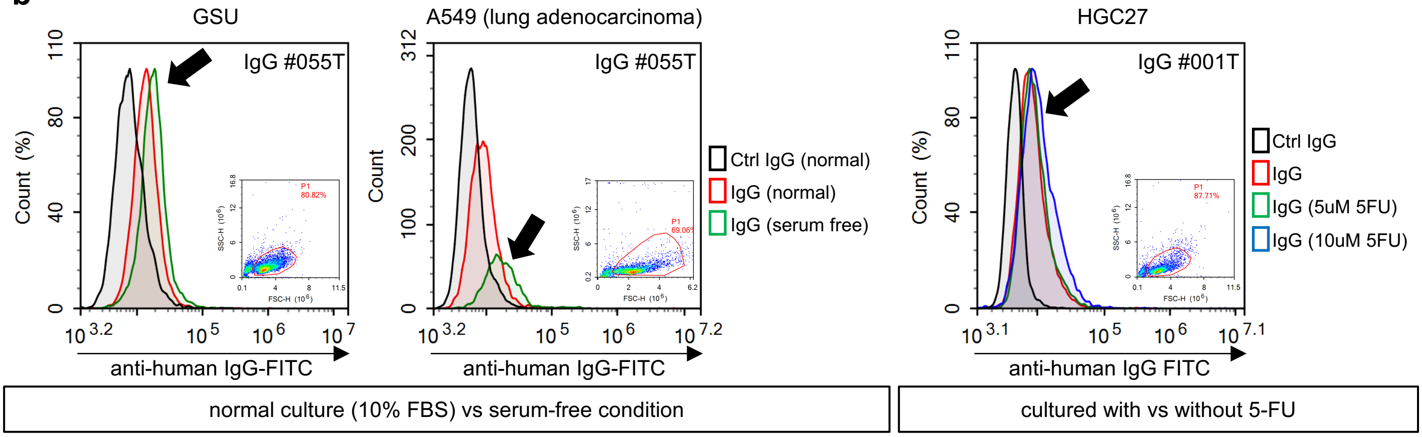

c

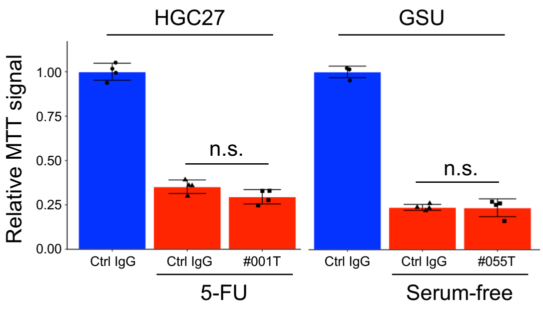

Supplementary Fig. 11: Biological functions of the human IgGs against FAK-related RNP antigens identified in this study. **a** MTT assays were performed to evaluate the anti-proliferative functions of the antibodies against various human GC cell lines *in vitro* (n=4). Asterisks indicate p<0.05 (t-test). The human IgG #013T exhibited very slight but significant growth suppressions in multiple GC cell lines. Experiments were performed at least in tetra-plicate and repeated at least twice. **b-c** Flow cytometry analyses were performed using our reconstructed IgGs and secondary FITC-conjugated goat anti-human IgG, and FITC signals were obtained after gating live cells via forward/side scatter plots (as exemplified by inserted graphs). It was revealed that a chemo-therapeutic agent and low-nutrition conditions, in some cases, resulted in enhanced exposures of the antigens to the outside of tumor cells; thus, such culture conditions induced increased reactivities of the antibodies to cancer cells (black arrows). Although MTT assays did not indicate significantly enhanced growth suppression of the antibodies in these conditions (t-test) (n=4) (**c**), it can be hypothesized that these antibodies could exhibit anti-tumor effects *in vivo* when combined with chemotherapies or under low-nutrition conditions of tumor microenvironments. MTT assays were performed in tetra-plicate and repeated twice.

Supplementary Fig. 12

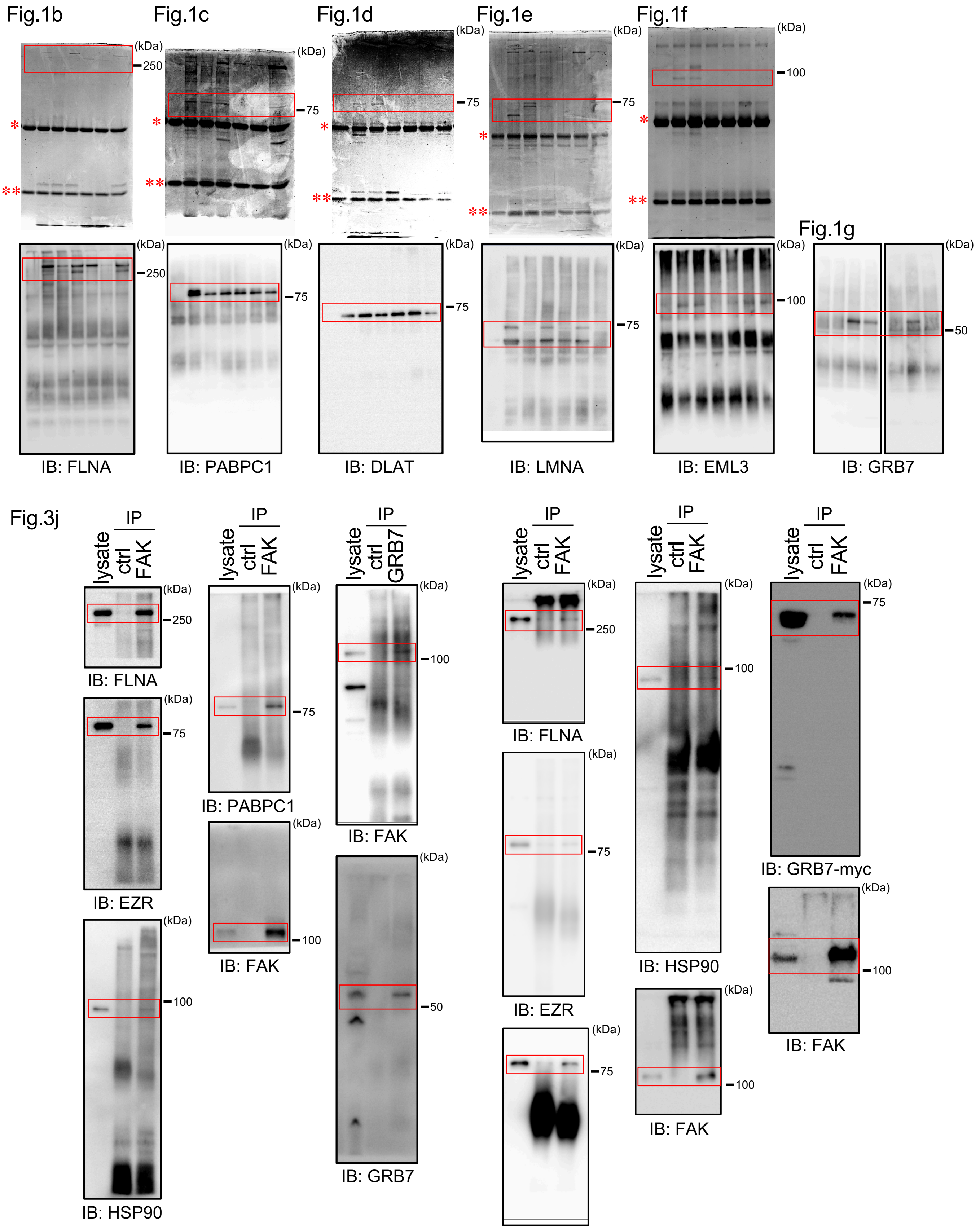

Supplementary Fig. 12: Uncropped blot/gel images for Fig. 1b-g, and Fig. 3j. Red squares indicate the cropped areas shown in the figures. Red asterisk; heavy chain of human IgG, red double-asterisk; light chain of human IgG.
