# Supplementary material for: Focal adhesion ribonucleoprotein complex proteins are major humoral cancer antigens and targets in autoimmune diseases
Source: Commun Biol. 2020 Oct 16;3:588. doi: 10.1038/s42003-020-01305-5 (PMC7567837; doi:10.1038/s42003-020-01305-5)
Supplement: Supplementary file 2 — Description of Additional Supplementary Files [file 42003_2020_1305_MOESM2_ESM.pdf]

Description of Additional Supplementary Files

File Name: Supplementary Data 1.
Description Precise clinicopathological factors of 102 GC cases analyzed in this study.

File Name: Supplementary Data 2.
Description Clinicopathological characteristics of 102 GC cohort analyzed in this study.

File Name: Supplementary Data 3.
Description List of the reconstructed human immunoglobulins (IgG1/k) and their identified antigens. The structures of the reconstructed human IgG1/k antibodies for which the protein antigens were identified are listed with full information on the V(D)J segments and amino acid sequences of CDRs. The left column represents names of the reconstructed antibodies with case IDs of the clinical GCs. Identified protein antigens are indicated in red.

File Name: Supplementary Data 4.
Description Identified peptides of the antigen proteins investigated in this study.

File Name: Supplementary Data 5.
Description NGS data set from the FAK-RIP experiments. The numbers of NGS sequence reads for all transcripts are shown for control (cellular total RNA) and biologically duplicated FAK-RIP experiments #1 and #2.

File Name: Supplementary Data 6.
Description FAK-RIP-enriched RNAs (more than five-fold enrichment compared to control) (log10 value). FAK-RIP-enriched RNAs (354 transcripts) are listed according to the rank of enrichments. * Pseudogenes were excluded from this list.

File Name: Supplementary Data 7.
Description GO enrichment analysis of the FAK-RIP-enriched RNAs. GO categories enriched among FAK-RIP-enriched RNAs (Supplementary Data 6) are listed. GO enrichment analysis was performed using the DAVID platform for Cellular Component, Biological Process, and Molecular Function. Enrichments of RhoGEF/RhoGAP families, Kinesin families, and endosome/lysosome membrane proteins are highlighted in red. P-values are shown as non-adjusted p-values calculated by the DAVID platform.
