# Supplementary material for: Focal adhesion ribonucleoprotein complex proteins are major humoral cancer antigens and targets in autoimmune diseases
Source: Commun Biol. 2020 Oct 16;3:588. doi: 10.1038/s42003-020-01305-5 (PMC7567837; doi:10.1038/s42003-020-01305-5)
Supplement: Supplementary file 4 — Reporting Summary [file 42003_2020_1305_MOESM4_ESM.pdf]

nature research

Corresponding author(s): Hiroto Katoh & Shumpei Ishikawa

Last updated by author(s): Sep 10, 2020

# Reporting Summary

Nature Research wishes to improve the reproducibility of the work that we publish. This form provides structure for consistency and transparency in reporting. For further information on Nature Research policies, see Authors & Referees and the Editorial Policy Checklist .

## Statistics

For all statistical analyses, confirm that the following items are present in the figure legend, table legend, main text, or Methods section.

| n/a | Confirmed | |
|---|---|---|
| ☐ | ☒ | The exact sample size (*n*) for each experimental group/condition, given as a discrete number and unit of measurement |
| ☐ | ☒ | A statement on whether measurements were taken from distinct samples or whether the same sample was measured repeatedly |
| ☐ | ☒ | The statistical test(s) used AND whether they are one- or two-sided<br>*Only common tests should be described solely by name; describe more complex techniques in the Methods section.* |
| ☐ | ☒ | A description of all covariates tested |
| ☐ | ☒ | A description of any assumptions or corrections, such as tests of normality and adjustment for multiple comparisons |
| ☐ | ☒ | A full description of the statistical parameters including central tendency (e.g. means) or other basic estimates (e.g. regression coefficient) AND variation (e.g. standard deviation) or associated estimates of uncertainty (e.g. confidence intervals) |
| ☐ | ☒ | For null hypothesis testing, the test statistic (e.g. *F*, *t*, *r*) with confidence intervals, effect sizes, degrees of freedom and *P* value noted<br>*Give P values as exact values whenever suitable.* |
| ☒ | ☐ | For Bayesian analysis, information on the choice of priors and Markov chain Monte Carlo settings |
| ☒ | ☐ | For hierarchical and complex designs, identification of the appropriate level for tests and full reporting of outcomes |
| ☒ | ☐ | Estimates of effect sizes (e.g. Cohen's *d*, Pearson's *r*), indicating how they were calculated |

*Our web collection on statistics for biologists contains articles on many of the points above.*

## Software and code

Policy information about availability of computer code

| | |
|---|---|
| Data collection | 2100 Expert (ver.B.02.08.ST648(SR2)), ImageLab Touch Software (ver.2.3.0.07), LAS AF (ver.3.1.0 build8587), Torrent Suite (ver.5.8.0) |
| Data analysis | Trimmomatic, Cutadapt, bwa, tximport package, TxDB, MEME suite, STAR software in Genomon RNA analysis pipeline, IgBlast, change-o, Alkazam software |

For manuscripts utilizing custom algorithms or software that are central to the research but not yet described in published literature, software must be made available to editors/reviewers. We strongly encourage code deposition in a community repository (e.g. GitHub). See the Nature Research guidelines for submitting code & software for further information.

## Data

Policy information about availability of data

All manuscripts must include a data availability statement. This statement should provide the following information, where applicable:

- Accession codes, unique identifiers, or web links for publicly available datasets
- A list of figures that have associated raw data
- A description of any restrictions on data availability

The BCR repertoire sequencing dataset generated during the current study is available in the Japanese Genotype-phenotype Archive (JGA)47 under an accession number JGAS00000000242. The RNA-seq dataset of the FAK-RIP experiment in this study is available in DDBJ Sequence Read Archive (DRA) under an accession number DRA010767.

# Field-specific reporting

Please select the one below that is the best fit for your research. If you are not sure, read the appropriate sections before making your selection.

# Life sciences study design

All studies must disclose on these points even when the disclosure is negative.

| | |
|---|---|
| Sample size | To delineate the global picture of anti-tumor humoral immunity, 102 tumor samples were collected and analyzed by repertoire sequencing. The theoretical diversity of humoral tumor antigens has been unpredictable; therefore, we collected as many clinical samples as possible. |
| Data exclusions | n/a |
| Replication | During the observations of the immunocytochemistry and immunofluorescent stainings, we carefully confirmed the reproducibility of our findings by observing multiple of cells at a time, as described in the text or figure legends. For the RNA-immunoprecipitation sequencing, two biological replicate samples were analyzed and only genes with reproducibility were further utilized for motif enrichment analysis. For biochemical and cell biological experiments, experiments were repeated at least twice as described in the text or figure legends. |
| Randomization | n/a |
| Blinding | n/a |

# Reporting for specific materials, systems and methods

We require information from authors about some types of materials, experimental systems and methods used in many studies. Here, indicate whether each material, system or method listed is relevant to your study. If you are not sure if a list item applies to your research, read the appropriate section before selecting a response.

## Materials & experimental systems

| n/a | Involved in the study | |
|---|---|---|
| ☐ | ☒ | Antibodies |
| ☐ | ☒ | Eukaryotic cell lines |
| ☒ | ☐ | Palaeontology |
| ☒ | ☐ | Animals and other organisms |
| ☐ | ☒ | Human research participants |
| ☒ | ☐ | Clinical data |

## Methods

| n/a | Involved in the study | |
|---|---|---|
| ☒ | ☐ | ChIP-seq |
| ☐ | ☒ | Flow cytometry |
| ☒ | ☐ | MRI-based neuroimaging |

## Antibodies

| | |
|---|---|
| Antibodies used | anti-FAK antibodies (#05-537, Merck Millipore; and AHO0502, Thermo Fisher Scientific), anti-GRB7 antibodies (PA5-79323, Thermo Fisher Scientific; and ab109618, Abcam), anti-FLNA antibody (HPA002925, Sigma-Aldrich), anti-PABP1 antibodies (#4992, Cell Signaling Technology; and ab21060, Abcam), anti-DLAT antibody (GTX109766, GeneTex), anti-LMNA antibody (ab108595, Abcam), anti-EML3 antibody (PA5-71024,Thermo Fisher Scientific), anti-EZR antibody (#07-130, Merck Millipore), anti-HSP90 antibody (#07-2174, Merck Millipore), anti-RPS6 antibody (#2217S, Cell Signaling Technologies), anti-Alpha II-spectrin antibody (A301-249A, Bethyl Laboratories), anti-CD138 antibody (#36-2900, Thermo Fisher Scientific), anti-CD20 antibody (ab78237, abcam), anti-CD4 antibody (ab133616, abcam), anti-CD68 antibody (ab955, abcam), anti-rabbit IgG Alexa-488 antibody (A-11008, Thermo Fisher Scientific), anti-mouse IgG Alexa-568 antibody (ab175701, Abcam). |
| Validation | All the antibodies were products of commercial vendors, and specificities, appropriate applications, and any related information were provided in their data sheets or websites of manufacturers. |

## Eukaryotic cell lines

Policy information about cell lines

| | |
|---|---|
| Cell line source(s) | GSU, HGC27, KatoIII, L23immo, MKN1, and Panc-1 were from RIKEN Bioresource Research Center (RIKEN BRC) (Japan), and A549, HL60, NUGC-3, and THP-1 were from Japanese Collection of Research Bioresources (JCRB) Cell Bank (Japan). |
| Authentication | Cell lines were authenticated by RIKEN BRC or JCRB. We have routinely checked the morphology of the cells under microscope. For GSU, HGC27, KatoIII, MKN1, NUGC3, A549, and Panc-1, we performed RNA-seq. For the L23immo cell line, immunofluorescent staining of a lineage marker was performed. |
| Mycoplasma contamination | Negativity for Mycoplasma infection has been regularly confirmed using TaKaRa PCR Mycoplasma Detection Set (TaKaRa Bio, Japan). |
| Commonly misidentified lines (See ICLAC register) | No commonly misidentified cell lines were used. |

## Human research participants

Policy information about studies involving human research participants

| | |
|---|---|
| Population characteristics | Frozen specimens of 102 gastric cancers that were surgically resected between 2009 and 2017 at the University of Tokyo Hospital were archived for this study under informed consent. |
| Recruitment | 102 gastric cancer cases were recruited for this study. Written informed consents were obtained from the patients. |
| Ethics oversight | This study was approved by the Institutional Review Boards of the University of Tokyo and Tokyo Medical and Dental University. |

Note that full information on the approval of the study protocol must also be provided in the manuscript.

## Flow Cytometry

### Plots

Confirm that:

- [x] The axis labels state the marker and fluorochrome used (e.g. CD4-FITC).
- [x] The axis scales are clearly visible. Include numbers along axes only for bottom left plot of group (a 'group' is an analysis of identical markers).
- [x] All plots are contour plots with outliers or pseudocolor plots.
- [x] A numerical value for number of cells or percentage (with statistics) is provided.

### Methodology

| | |
|---|---|
| Sample preparation | Cells were cultured under conditions as indicated in Supplementary Fig. 11; then, cells were incubated with our reconstructed immunoglobulins. Alexa-488 conjugated goat anti-human IgG antibody (A11013, invitrogen) was used as a secondary antibody. |
| Instrument | NovoCyte (ACEA Bioscience, CA, USA) |
| Software | NovoExpress (ACEA Bioscience, CA, USA) |
| Cell population abundance | Cell sorting was not performed. |
| Gating strategy | Select live cell populations via forward/side scatter plots, and no further gating was performed. |

- [x] Tick this box to confirm that a figure exemplifying the gating strategy is provided in the Supplementary Information.
